# The Synthetic Cannabinoid ADB-FUBINACA Disrupts Mitochondrial Morphology and Dynamics during Neuronal Differentiation of NG108-15 Cells

**DOI:** 10.1007/s12035-026-05699-x

**Published:** 2026-01-21

**Authors:** Rui Filipe Malheiro, Ana Catarina Costa, Catarina Pereira-Teixeira, Helena Carmo, Félix Carvalho, João Pedro Silva

**Affiliations:** 1 Associate Laboratory Institute for Health and Bioeconomy (i4HB), Faculty of Pharmacy, University of Porto, Rua Jorge de Viterbo Ferreira 228, 4050-313 Porto, Portugal; 2 Applied Molecular Biosciences Unit (UCIBIO), Laboratory of Toxicology, Department of Biological Sciences, Faculty of Pharmacy, University of Porto, Rua Jorge de Viterbo Ferreira 228, 4050-313 Porto, Portugal; 3 Nerve Regeneration Group, Instituto de Biologia Molecular E Celular (IBMC), Instituto de Investigação E Inovação Em Saúde (i3S), University of Porto, Rua Alfredo Allen 208, 4200-135 Porto, Portugal

**Keywords:** Neurodevelopment, New Psychoactive Substances, Mitochondrial fission and fusion, Mitochondrial network, Mitochondrial transport

## Abstract

Mitochondria are essential drivers of neuronal growth, differentiation, and overall brain development. Synthetic cannabinoids (SCs) have been shown to enhance neurite outgrowth in NG108-15 neuroblastoma x glioma cells through CB1 receptor activation, while disrupting mitochondrial function. Here, we demonstrated first-hand the impact of biologically-relevant concentrations (< 1μM) of ADB-FUBINACA (an SC commonly identified in drug seizures) on mitochondrial morphology and dynamics (i.e., fusion, fission and mobility) during the neurodifferentiation of NG108-15 cells. Our findings revealed that, during NG108-15 neurodifferentiation, ADB-FUBINACA reduced the mean mitochondrial area and perimeter by around 10%, while increasing mitochondrial circularity, and decreasing network branching and interconnectivity. Specifically, branch length per mitochondrion and branch junctions declined by 17 and 25% in the neurons’ soma at the end of NG108-15 differentiation (after 72 h). Moreover, 1 nM and 1 µM ADB-FUBINACA markedly decreased the levels of mitochondrial fusion markers (Opa1 and Mfn2) and increased the levels of fission markers Drp1 and Fis1 at the same time point. The percentage of motile mitochondria in neurites also decreased at 72 h, while average speed and total run length per mobile mitochondrion remained unaffected, resulting in an accumulation of stationary mitochondria which may be important, for example, to support neurite extension. Collectively, these findings suggest that while ADB-FUBINACA promotes mitochondrial accumulation in neurites, potentially supporting the energy demands of developing neurites and influencing neurite outgrowth, in the long-term, the fragmentation of the mitochondrial network in the soma may compromise the maintenance of neurites, in terms of energy requirements.

**Supplementary Information:**

The online version contains supplementary material available at 10.1007/s12035-026-05699-x.

## Introduction

The pivotal role played by mitochondria during neuronal stem cell reprogramming and differentiation has received increasing attention over the past few years [1]. Neurons rely on mitochondrial activity for processes including ATP production, maintenance of redox balance, calcium signalling, and regulation of gene expression, all of which being essential for neurite growth, dendrite remodelling, and neurotransmitter release [2]. The differentiation of stem cells into a specific neuronal lineage involves a metabolic shift, marked by increased mitochondrial respiration, and significant changes in mitochondrial dynamics, including alterations in their shape and network structure [3]. In addition, an efficient distribution and strategic positioning of mitochondria is crucial to support specialized functions in various cellular regions. For instance, during neurite outgrowth, mitochondria must be actively transported to the distal ends of neurites, far from the cell body, where they supply the high levels of energy required for growth and other specialized functions [4, 5].

Notably, there is accumulating evidence suggesting that the modulation of the endocannabinoid system, particularly via CB1 receptor signalling, can influence mitochondrial activity [6–10]. For example, Bénard et al. demonstrated the presence of mitochondrial CB1 receptor (mtCB1) in the membranes of neuronal mitochondria, and showed that the activation of mtCB1 controlled cellular respiration and energy production [11]. Additionally, Hebert-Chatelain et al. (2016) showed that activation of mtCB1 in hippocampal neurons resulted in a reduction in the number of mobile axonal mitochondria [12].

Moreover, the endocannabinoid system’s components can be detected from the earliest stages of embryonic development [13]. For example, the expression of CB1 receptors has been observed in neuronal populations during early embryonic development, even before the formation of synapses, seemingly correlating with neuronal differentiation [14]. As such, it is reasonable to expect that exogenous cannabinoids, which target the endocannabinoid system, play a modulatory role in neurogenesis, as highlighted by both *in vitro* [15–17] and *in vivo* [18, 19] studies. For example, SCs such as AMB-FUBINACA, ADB-FUBINACA [20], and THJ-2201 [21] have been shown to enhance the neurite outgrowth in NG108-15 neuroblastoma x glioma hybrid cells in a process mediated through the activation of CB1 receptors. Notably, the use of SCs remains a major public health issue, driven by their easy availability and higher potency compared to cannabis. This concern is heightened by their growing use among young adults. In particular, pregnant and lactating women, and women of childbearing age, represent major risk groups, given SCs’ ability to cross the placenta and accumulate in fetal tissues, potentially impacting development [22].

While a growing body of research has examined the role of the endocannabinoid system [23] and mitochondrial function during neurodifferentiation [24–27], the implications of such an interplay to neuronal differentiation remain poorly understood [26, 28]. Here, we build up on our previous findings to explore the contribution of mitochondrial dynamics (e.g., fusion, fission, and mobility) to ADB-FUBINACA-induced promotion of neurite outgrowth in NG108-15 cells.

Exploring how SCs interfere with the endocannabinoid system’s modulation of mitochondrial dynamics is fundamental for a deeper understanding of how SCs may influence neuronal differentiation, hence contributing to assessing the risk of these substances to young adults.

## Materials and Methods

### Chemicals

Heat-inactivated fetal bovine serum (FBS), antibiotic solution (10,000 U/mL penicillin, 10,000 µg/mL streptomycin), 0.25% trypsin/EDTA and Hank’s Balanced Salt Solution with calcium and magnesium (HBSS) were obtained from PAN-Biotech (Aidenbach, Germany). PKmito RED probe was supplied by Spirochrome (Stein am Rhein, Switzerland). All other reagents used in this study were obtained from Merck (Darmstadt, Germany), unless otherwise specified.

### Synthetic Cannabinoid

(S)-N-(1-amino-3,3-dimethyl-1-oxobutan-2-yl)−1-(4-fluorobenzyl)−1H-indazole-3-carboxamide (ADB-FUBINACA) was provided by TicTac Communications Ltd, UK. ADB-FUBINACA replicates the pharmacological effects of Δ^9^-tetrahydrocannabinol (Δ^9^-THC) with up to 85 and 140 times more potency, presenting a half-maximal effective concentration (EC_50_) of 1.2 nM and 3.5 nM at CB1 and CB2, respectively [29]. Stock solutions of ADB-FUBINACA (5 mM) were prepared in dimethyl sulfoxide (DMSO). Prior to cell exposure, these stock solutions were serially diluted in HBSS to achieve a final DMSO concentration below the threshold known to induce NG108-15 cell differentiation under low serum conditions (i.e., below 0.1%) [30].

### Cell Culture

The mouse neuroblastoma × rat glioma NG108-15 cell line, sourced from the European Collection of Authenticated Cell Cultures (ECACC, Salisbury, UK), was cultured according to established protocols [21]. Cells were grown in 75 cm^2^ flasks containing Dulbecco’s Modified Eagle’s Medium (DMEM), supplemented with 10% (v/v) heat-inactivated FBS and an antibiotic solution comprising 100 U/mL penicillin and 100 µg/mL streptomycin. Cell cultures were maintained at 37° C in a humidified atmosphere with 5% CO_2_. Upon reaching 80–90% confluence, cell detachment for subculturing or seeding was carried out using a 0.25% trypsin–EDTA solution. Differentiation of NG108-15 cells into cholinergic neurons followed a well-established protocol [21], comprising the replacement of the complete cell culture medium (hereafter referred to as maintenance medium) with DMEM supplemented with 1% FBS, 10 µM retinoic acid, and 30 µM forskolin (hereafter denoted as differentiation medium). ADB-FUBINACA exposure was performed at biologically-relevant concentrations (ranging from 1 pM to 1 µM) immediately after the replacement of the maintenance medium by differentiation medium. Noteworthy, within a span of 72 h, cells undergo morphological changes, transitioning from polygonal flattened bodies to oval shapes, while developing cholinergic traits and forming a distinct neurite network (Supplementary Information, Figure SI-1), serving as a reliable model for studying neuritogenesis and synaptogenesis, crucial processes in neuronal development [31]. Importantly, NG108-15 cells inherently express the CB1 receptor, providing an added advantage for studying the neurotoxic effects of cannabinoids.

### Assessment of Mitochondrial Morphology and Mobility

Quantitative analysis of mitochondrial morphology and mobility was conducted using fluorescence confocal microscopy and 2D image processing [32]. NG108-15 cells were seeded at a density of 1.0 × 10^4^ cells per well in 8-well chamber slides (Ibidi, Germany). After an overnight incubation period, neurodifferentiation was initiated, and the cells were exposed in parallel to ADB-FUBINACA (1 pM – 1 μM) or 0.1% DMSO (vehicle control) for 24 or 72 h. At each timepoint, the cells were incubated (37 °C, 5% CO_2_) with differentiation medium containing PKmito RED (1:3000) for 30 min. Notably, PKMito Red relies on membrane potential for accumulation, which restricts labelling of depolarized mitochondria. Nevertheless, although not covalently bound, PKMito Red remains strongly retained within mitochondria, enabling extended imaging [33]. The medium containing the probe was then aspirated and replaced with fresh medium. A condition in which cells were incubated for 15 min with 10 μM carbonyl cyanide m-chlorophenyl hydrazone (CCCP) before probe loading was also included (positive control). CCCP, a potent mitochondrial uncoupler, is known to induce significant morphological changes in mitochondria, such as fragmentation of the mitochondrial network [34, 35]. Neuronal cell live-imaging was performed using a fluorescence confocal microscope (Nikon Crest X-Light V3) equipped with a 60 × objective, in combination with DeepSIM X-Light super-resolution system.

During imaging, the cells were maintained at 37° C and 5% CO₂ on the microscope stage. To analyse mitochondrial morphology, images were processed using the Mitochondria Analyzer plugin from ImageJ/Fiji software[36] (Supplementary Information, Figure SI-2). Various parameters were assessed, including the number of mitochondria/networks, mitochondrial area, perimeter, aspect ratio, form factor, number of branches, branch length, and branch junctions. Time-lapse series of image stacks were captured every 1.7 s over a span of 10 min to evaluate the mitochondria transport along neurites [37]. Maximum intensity Z-projections were generated for each time point. To ensure accurate tracking, 100 μm neurite segments starting at least 20 μm from the soma were selected. The MTrackJ plugin was used to track individual mitochondrion in the image sequences and perform data statistics. Mitochondria exhibiting movement of at least 20 μm from their initial location over 10 min were considered motile.

### Quantification of Protein Levels

#### Total Protein Extraction

Total protein extraction, intended for subsequent analysis by Western blot, was carried out following a protocol previously described [21]. Cells were seeded in 6-well plates at a density of 3.0 × 10^5^ cells per well and allowed to adhere overnight. Total protein was extracted either before the initiation of differentiation (i.e., in undifferentiated cells) or at 24-, 48-, and 72 h after neurodifferentiation was initiated, and ADB-FUBINACA was added. The cell culture medium was aspirated, and the cells were detached in HBSS using a cell scraper, and then transferred to 15 mL tubes. Subsequently, the cell suspensions were centrifuged at 1000 × g, 4ºC for 5 min (Eppendorf® centrifuge 5810 R, Hamburg, Germany). After removing the supernatants, the pellets were resuspended in 1 mL HBSS and recentrifuged under the same conditions. The supernatants were once again discarded, and the pellets were resuspended in 100 µL of collection buffer (20 mM HEPES, 250 mM sucrose, 10 mM KCl, 2 mM MgCl_2_, 1 mM EDTA, pH 7.5), supplemented with 1 mM sodium orthovanadate, 2 mM dithiothreitol (DTT), 100 µM phenylmethylsulfonyl fluoride (PMSF), and Protease Inhibitor Cocktail (P8340, Sigma-Aldrich). For cell disruption, samples underwent sonication with three 10-s pulses at 30% amplitude interspersed with 30-s intervals on ice. Protein concentrations from each sample were quantified using the Bio-Rad Detergent Compatible (DC) protein assay as per the manufacturer’s instructions, and subsequently stored at −80ºC until required.

#### Western-Blot

Protein levels was assessed through Western blot on total cell extracts, following a well-established procedure with minor adjustments[21]. Sample protein extracts (40 µg) were denatured in sample loading buffer (0.25 M Tris–HCl, 50% glycerol, 10% sodium dodecyl sulfate (SDS), 0.2 M DTT, and 0.001% bromophenol blue) at 95 °C for 5 min. Electrophoresis was conducted on 10–15% SDS–polyacrylamide gels, followed by transfer to polyvinylidene fluoride (PVDF) membranes (GE Healthcare, Pittsburgh, PA, USA) using the Trans-Blot Turbo Transfer System (Bio-Rad, Hercules, CA, USA) at 25 V for 30 min. The membranes were subsequently blocked with 5% skimmed milk prepared in phosphate-buffered saline containing 0.05% Tween 20 (TPBS), pH 7.4, for 2 h at room temperature. After three 10-min washes with TPBS, the membranes were incubated overnight at 4° C with primary antibodies: mouse anti-Drp1 (1:200, sc-271583), mouse anti-Mfn1 (1:100, sc-166644), mouse anti-Mfn2 (1:400, sc-100560), mouse anti-Miro1 (1:200, sc-398520), mouse p-Tau antibody (1:200, sc-32275), mouse Tau (1:200, sc-21796, Santa Cruz Biotechnology, CA, USA), mouse Opa1 (1:1000, Sigma-Aldrich, St. Louis, MO, USA), rabbit anti-Phospho-DRP1 (Ser616) (1:500, Invitrogen PA5-64,821), rabbit anti-MiD51 (1:1000, Invitrogen PA5-99,970) and rabbit anti-Fis1 (1:2000, Proteintech, IL, USA). Samples were additionally probed with mouse anti-β-actin antibody (1:4000, Sigma-Aldrich, St Louis, MO, USA) to normalize the data obtained. Primary antibodies were diluted in a 1% BSA solution prepared in TPBS and supplemented with 0.05% sodium azide. The membranes were washed three times with TPBS for 10 min each, before being incubated with horseradish peroxidase-conjugated anti-mouse IgG (1:2500) or anti-rabbit IgG (1:1000, Advansta, CA, USA), prepared in a 1% BSA/TPBS solution, for 1 h at room temperature. The chemiluminescence reaction was initiated by adding the Clarity Western ECL detection reagent (GE Healthcare, Chicago, USA) and the signals detected on a molecular imager ChemiDoc™ XRS (Bio-Rad, Hercules, CA, USA). Band intensities for each protein were quantified using Image Lab, version 6.0 (Bio-Rad, Hercules, CA, USA), and normalized to the intensities of the corresponding β-actin bands. Subsequently, the results were expressed as the relative levels of each protein in comparison to the vehicle control at 24 h.

### Statistical Analysis

Statistical analysis was performed using GraphPad Prism 8 software (GraphPad Software, La Jolla, CA, USA). The normality of each distribution was assessed through the Anderson–Darling, D’Agostino–Pearson, and Shapiro–Wilk normality tests, taking into account the acceptability of skewness and kurtosis values. The tests employed, the number of independent experiments and the number of replicates (if applicable) are specified in the figure legends.

## Results

### ADB-FUBINACA Disrupted Mitochondrial Structure and Interconnectivity in Neuronal Soma-located Mitochondria

Cells were exposed to ADB-FUBINACA for 24 and 72 h to assess its impact on mitochondrial network morphology and connectivity, followed by PKMito Red staining and live-cell imaging. Separate analyses were performed for mitochondria in the soma and those in the neurites to thoroughly evaluate how ADB-FUBINACA affects mitochondrial dynamics in different cellular compartments.

**Fig. 1:**
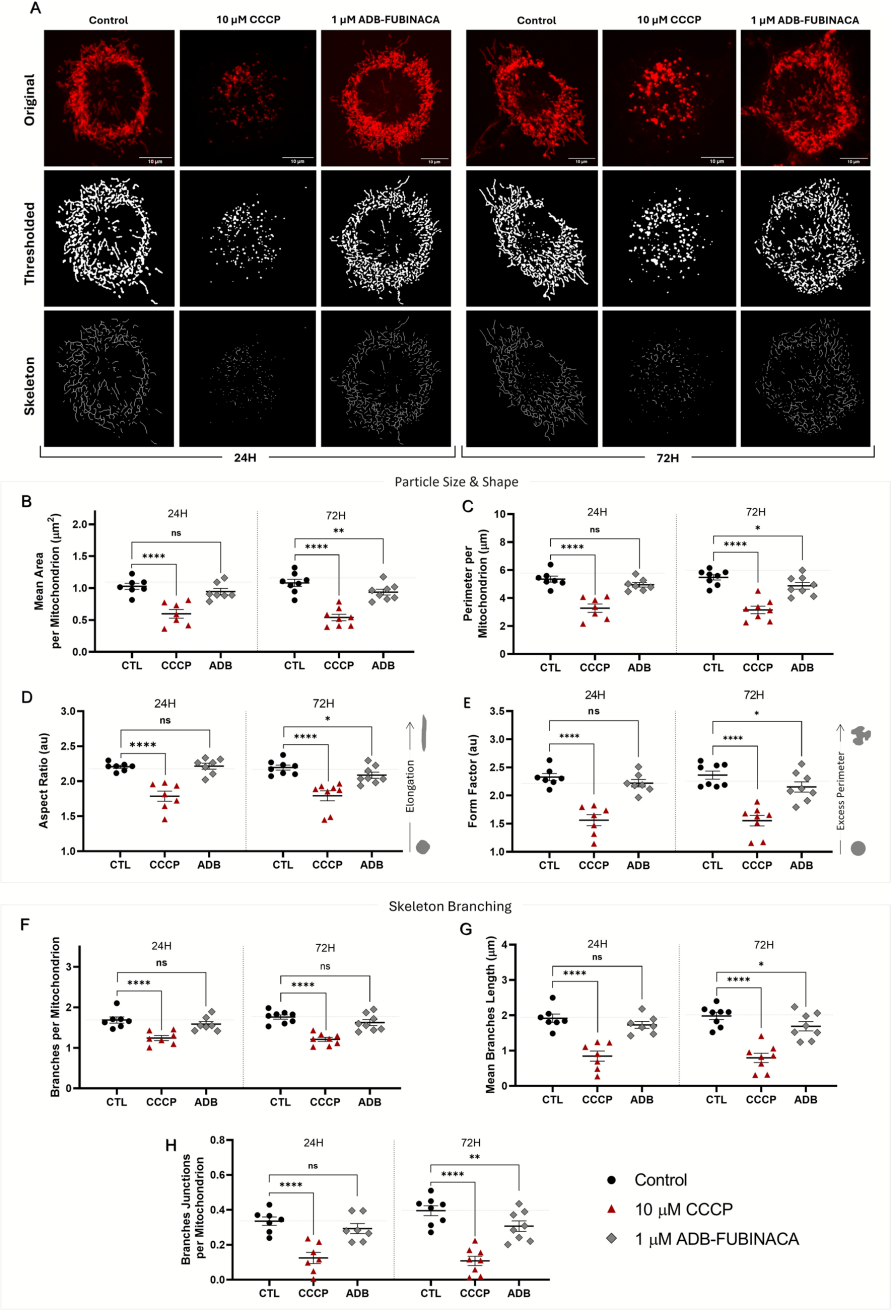
Assessment of mitochondrial morphology in the soma of NG108-15 cells, following 24 and 72 h treatment with 1 µM ADB-FUBINACA. (**A**) The mitochondrial network images stained with the PKMito RED probe are presented in their raw format as well as following thresholding and skeletonization processing. Images were quantified and analyzed with the Mitochondria Analyzer Plugin from Fiji (ImageJ), enabling the assessment of various parameters such as (**B**) mean mitochondrial area, (**C**) perimeter, (**D**) aspect ratio, (**E**) form factor, (**F**) number of branches per mitochondrion, (**G**) mean branch length, and (**H**) number of branch junctions per mitochondrion. Results are presented as mean ± SEM, based on at least seven independent experiments. Between 7 and 14 cells were analysed per condition in each independent experiment, resulting in a minimum of 70 cells evaluated per condition across all experiments. *p < 0.05, **p < 0.01, compared to the respective vehicle control (72 h), using Nested one-way ANOVA, followed by Dunnett’s post-test

As shown in Fig. 1A, mitochondria in the soma from control neurons, both at 24 and 72 h, were observed as either separate and distinctly shaped small organelles or as part of a complex and interconnected network with extensive branching. Notably, no changes in this network were observed between these time points. Nonetheless, exposure to CCCP for 15 min resulted in severe mitochondrial fragmentation, producing smaller, spherical mitochondrial units, consistent with previous reports [34, 35, 38–40]. This fragmentation was evident for all analysed endpoints, such as mitochondrial area, perimeter, shape, and network branching, highlighting CCCP's profound impact on mitochondrial structure and function, thereby validating our method.

Notably, 1 µM ADB-FUBINACA disrupted mitochondrial morphology and connectivity (i.e. network branching) in the neuronal soma after 72 h exposure, with no noticeable effects observed at 24 h. In particular, ADB-FUBINACA reduced the mean mitochondrial area per mitochondrion (0.91 ± 0.05 µm^2^ vs. 1.05 ± 0.06 µm^2^ in control, Fig. 1B), suggesting a more condensed structure or possible content loss at 72 h. The mean perimeter also decreased in the presence of the SC (4.88 ± 0.24 µm vs. 5.48 ± 0.19 µm in controls, Fig. 1C), reflecting the formation of smaller, more compact mitochondrial structures [41]. Additionally, exposure to ADB-FUBINACA also altered mitochondrial shape, as shown by the reduction in aspect ratio (a measure of mitochondrial elongation) and form factor (quantification of how closely the shape of mitochondria gets of a perfect circle) (Figs. 1D and 1E, respectively). The aspect ratio decreased from 2.20 ± 0.04 in control to 2.09 ± 0.04 in ADB-FUBINACA-exposed cells, while the form factor decreased from 2.36 ± 0.07 in the control to 2.15 ± 0.09 in SC-treated cells. Together, these changes indicate a shift from elongated to more spherical and compacted forms over the differentiation period.

We further analysed mitochondrial branching due to its critical role in establishing an interconnected network that supports efficient energy production and the proper distribution of mitochondrial contents. As represented in Fig. 1F, the number of branches per mitochondrion remained unchanged after 72 h exposure to ADB-FUBINACA (1.62 ± 0.05 vs 1.76 ± 0.07 in control). However, the average branch length was significantly reduced in ADB-exposed cells (1.69 ± 0.13 µm) compared to the control (1.98 ± 0.10 µm), as depicted in Fig. 1G. Additionally, the number of branch junctions decreased (0.31 ± 0.03 vs. 0.40 ± 0.08 in control, Fig. 1H), indicating a loss of mitochondrial connectivity.

### ADB-FUBINACA Led to the Accumulation of Mitochondria Along Neurites

**Fig. 2:**
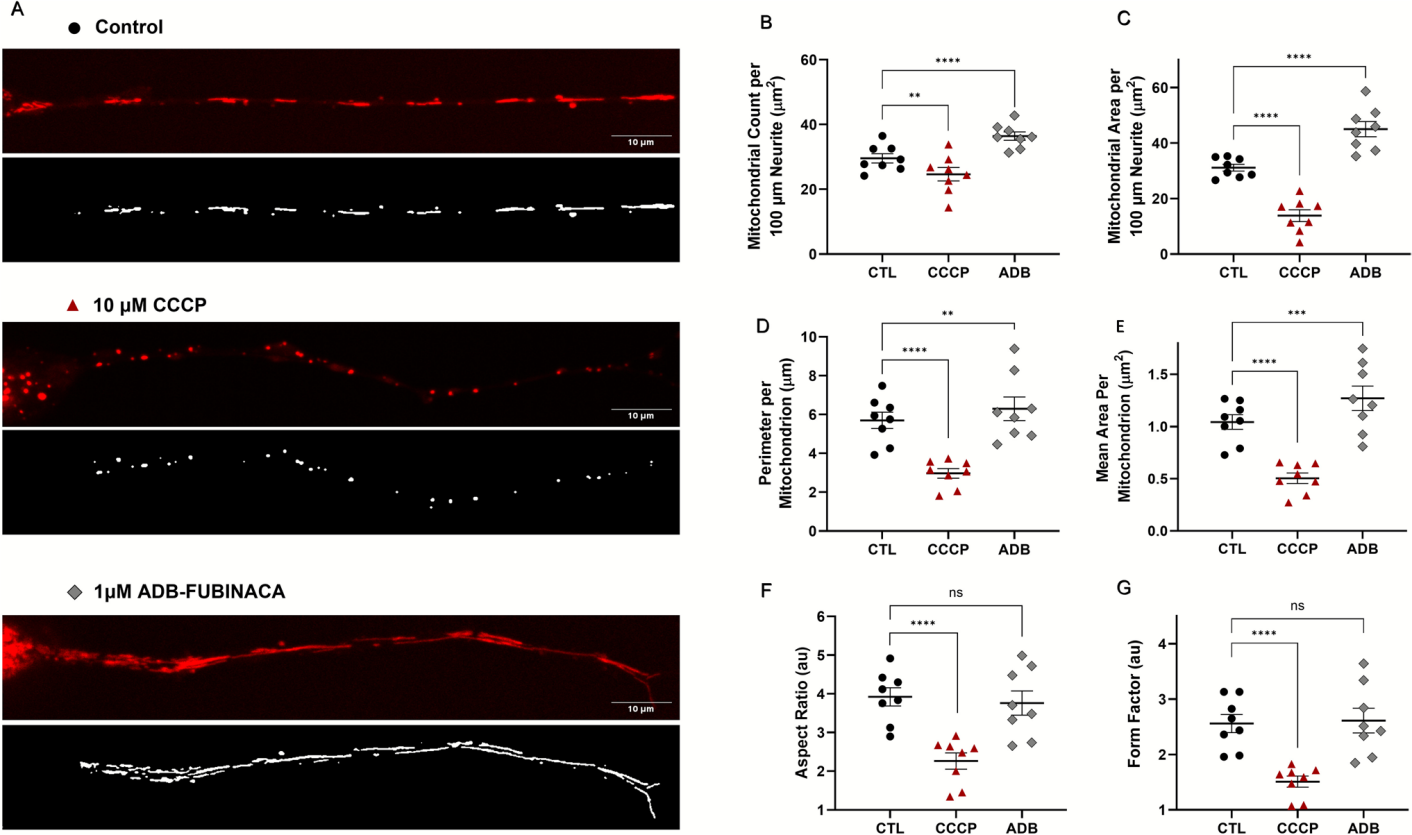
Evaluation of mitochondria in neurites after 72 h exposure to 1 µM ADB-FUBINACA. (**A**) Representative images depicting the mitochondrial network in neurons stained with the PKMito RED probe, displayed in their raw form and after thresholding. (**B**) Quantification of Mitochondrial Number and (**C**) Total Mitochondrial Area per 100 µm length of neurite. (**D**) Measurement of Mitochondrial Perimeter and (**E**) Area per mitochondrion. Assessment of mitochondrial shape profile, including (**F**) Aspect Ratio and (**G**) Form Factor. Results are presented as mean ± SEM from seven independent experiments, with a minimum of 61 neurites assessed per condition (ranging from 5 to 18 neurites per experiment), totaling 339 neurites analyzed. ****p < 0.0001 compared to the respective vehicle control, using Nested one-way ANOVA, followed by Dunnett’s post-test

Given the high energy demands of neurite outgrowth, we also ascertained whether ADB-FUBINACA interfered with mitochondrial dynamics within neurites. This assessment was carried out at 72 h, when neurite extensions were sufficiently developed for a comprehensive analysis. Under control conditions, mitochondria typically display tubular or ovoid structures, distributed along the entire length of the neurites (Fig. 2A, top image). As expected, treatment with CCCP resulted in mitochondrial fragmentation, reducing their overall area and number within the neurite, as observed in Fig. 2A, center image. Notably, 1 µM ADB-FUBINACA increased both the number and area of mitochondria within neurites, compared to the vehicle control (Figs. 2B and 2 C, respectively). Specifically, the mitochondrial count per 100 µm-segment increased from 29.55 ± 1.42 in control cells to 36.44 ± 1.29 in ADB-FUBINACA-treated cells, whereas total mitochondrial area increased from 31.11 ± 1.22 µm^2^ in control cells to 45.02 ± 2.73 µm^2^ in ADB-FUBINACA-treated cells, indicating a substantial enhancement of mitochondrial retention within neurites. Notably, the average perimeter per mitochondrion increased from 5.69 ± 0.42 µm in control cells to 6.29 ± 0.61 µm in ADB-FUBINACA-treated cells (Fig. 2D), while the area per mitochondrion increased from 1.04 ± 0.07 µm^2^ in control cells to 1.27 ± 0.12 µm^2^ (Fig. 2E), suggesting an overall enlargement of the mitochondria. Despite this, no alterations in mitochondrial shape were detected following exposure to the SC, as reflected by the unchanged aspect ratio (3.76 ± 0.31 vs. 3.92 ± 0.24 in control, Fig. 2F) and form factor (2.61 ± 0.22 vs. 2.56 ± 0.16 in the control, Fig. 2G).

### ADB-FUBINACA Reduced the Levels of Mitochondrial Fusion Markers

We also analyzed whether ADB-FUBINACA altered the levels of different markers of mitochondrial fusion and fission in differentiating NG108-15 cells, given that the balance between these processes is key to regulate mitochondrial shape and size, and to maintain mitochondrial structure. In particular, we targeted three key dynamin family GTPases fusion-related proteins: Optic atrophy-1 (Opa1), which is crucial for inner membrane fusion, and Mitofusins 1 (Mfn1) and 2 (Mfn2), which regulate outer membrane fusion [42].

**Fig. 3:**
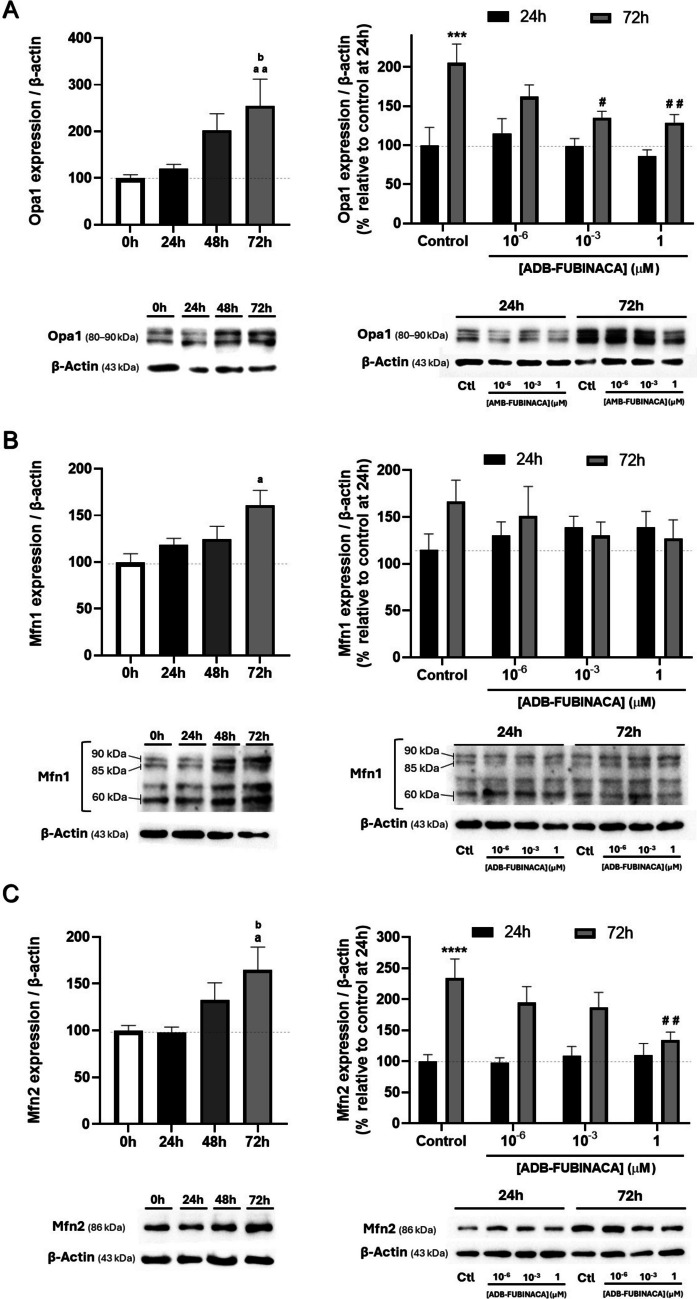
Impact of ADB-FUBINACA on Mitochondrial Fusion during neurodifferentiation. Analysis of (**A**) Opa1 (80–90 kDa), (**B**) Mfn1 (60, 85, and 90 kDa), and (**C**) Mfn2 (86 kDa) levels during 72 h of NG108-15 cell differentiation and the impact of ADB-FUBINACA after 24 and 72 h, using Western blot. Protein bands were normalized by the amount of β-actin in the respective lane. Data are presented as mean ± SEM from at least six independent experiments. ^a^p < 0.05, ^aa^p < 0.01, compared to vehicle-control at 0 h. ^b^p < 0.05, compared to vehicle-control at 24 h, using one-way ANOVA followed by Tukey’s post-hoc test. ***p < 0.001, ****p < 0.0001 compared to vehicle-control at 24 h. ^#^*p* < 0.05, ^##^*p* < 0.01, compared to vehicle-control at 72 h, using two-way ANOVA followed by Dunnett’s post-hoc test

We observed a gradual increase in the levels of all these markers throughout NG108-15 differentiation, with highest levels being attained at 72 h (differentiated cells), as noted in Fig. 3. Specifically, Opa1, Mfn1, and Mfn2 levels in the control increased by around 2.5, 1.6, and 1.7-fold, respectively, compared to pre-differentiation levels, underscoring an enhancement of the mitochondrial fusion machinery. However, exposure to ADB-FUBINACA resulted in the cells’ failure to sustain the increase in Opa1 and Mfn2 levels observed in control cells. Specifically, at 72 h, Opa1 levels were about 60–65% lower following 1 nM and 1 µM ADB-FUBINACA exposure, compared to the control (Fig. 3A), while Mfn2 levels decreased by 57% at 1 µM (Fig. 3C) compared to the respective control. The SC did not seem to change any of these mitochondrial fusion markers 24 h after exposure. Notably, Mfn1 levels remained unchanged by ADB-FUBINACA at both 24 and 72 h.

Noteworthy, OPA1 was detected as five major isoforms ranging from 80 to 90 kDa. This aligns with reports of Opa1 undergoing alternative splicing, and generating eight mRNAs that produce five major protein isoforms within the referred molecular weight range (80–90 kDa) [43]. MFN1 appeared as three distinct bands at approximately 85 kDa, 90 kDa, and 60 kDa, which can also be attributed to this protein’s different isoforms and that have been previously detected by others [44].

### ADB-FUBINACA Increased the Levels of Proteins Involved in Mitochondrial Fission

Analysis of the impact of ADB-FUBINACA on mitochondrial fission mainly focused on four key proteins: Dynamin-related protein 1 (Drp1) and its phosphorylated form (p-Drp1) at Ser616, the primary regulator of mitochondrial fission, which assembles at construction sites to facilitate the division of mitochondria; Fis1 (Mitochondrial Fission 1), a tail-anchored protein in the mitochondrial outer membrane crucial for recruiting Drp1; and MiD51 (or SMCR7L), an adaptor protein that facilitates Drp1 assembly [42].

**Fig. 4:**
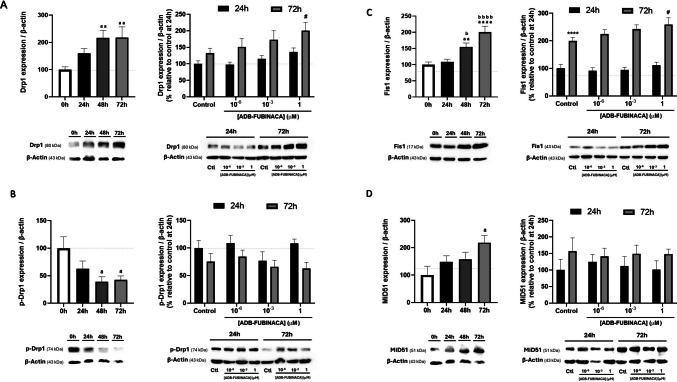
Evaluation of mitochondrial fission markers levels following exposure to ADB-FUBINACA. Quantification of (**A**) Drp1 (80 kDa), (**B**) p-Drp1 (74 kDa), (**C**) Fis1 (17 kDa) and (**D**) MiD51 (51 kDa) was performed by Western blot over a 72-h period to evaluate their expression profiles during NG108-15 cells differentiation and the effects of ADB-FUBINACA evaluated following 24 and 72 h of exposure. The density of bands was normalized by the amount of β-actin per lane. Each bar represents the mean ± SEM, for at least five independent experiments. ^aa^p < 0.01, ^aaaa^p < 0.0001 compared to vehicle-control at 0 h. ^b^p < 0.05, ^bbbb^p < 0.0001 compared to vehicle-control at 24 h, using one-way ANOVA followed by Tukey’s post-hoc test. ****p < 0.0001, compared to vehicle-control at 24 h. ^#^*p* < 0.05 compared to vehicle-control at 72 h, using two-way ANOVA followed by Dunnett’s post-hoc test

The levels of mitochondrial fission markers increased during NG108-15 differentiation in the control. As illustrated in Fig. 4A (left), Drp1 levels showed a significant increase, reaching a maximum at 48 h that persisted until 72 h. Fis1 and MiD51 levels displayed a gradual increase over time, reaching a twofold increase at 72 h compared to pre-differentiated cells (Fig. 4C and 4D). However, the phosphorylated form of Drp1, which represents the fission-competent state, decreased over the same period (Fig. 4B). This indicates that although Drp1 abundance was higher, the protein was not being converted into its active form. Exposure to 1 µM ADB-FUBINACA increased the levels of both fission proteins at 72 h, with no notable changes observed at 24 h. Specifically, Drp1 levels increased by 1.5-fold, while Fis1 levels rose by approximately 1.3-fold in the presence of 1 µM ADB-FUBINACA, compared to their respective controls, as shown in Figs. 4A and 4B (right). Meanwhile, MiD51 and p-Drp1 levels were not affected by ADB-FUBINACA exposure (Figs. 4B and 4D).

### ADB-FUBINACA Increased the Percentage of Static Mitochondria in Neurites

**Fig. 5:**
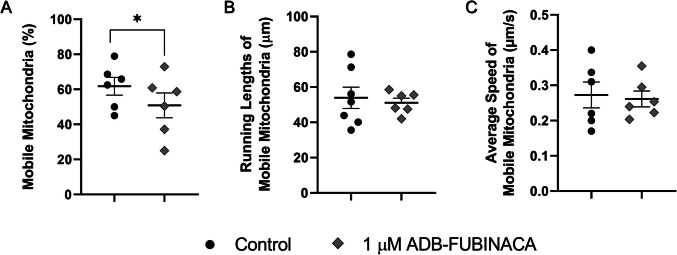
Assessment of mitochondrial trafficking in NG108-15 cell neurites, labelled with PKmito RED. Monitoring and tracking of mitochondrial movement along 100 μm neurite segments over 10 min, using MTrackJ plugin. (**A**) Percentage of mobile mitochondria, defined as those showing movement of 20 μm or more. (**B**) Mean path length for each mobile mitochondrion. (**C**) Average speed of mobile mitochondria. Each bar represents the mean ± SEM, for at least six independent experiments. *p < 0.05, compared to vehicle-control, using Nested one-way ANOVA, followed by Dunnett’s post-test

The increased number of mitochondria within neurites may suggest changes in mitochondrial mobility and trafficking. As such, we evaluated the movement of individual mitochondria in neurites at 72 h, when neurite extensions were sufficiently developed to allow the comprehensive tracking of mitochondrial movement across extensive lengths. Exposure of NG108-15 cells to 1 µM ADB-FUBINACA reduced mitochondrial movement, with the percentage of motile mitochondria dropping to 50.88 ± 7.08%, compared to 61.82 ± 5.10% in the control (Fig. 5A), as observed in the supplementary video. Notably, ADB-FUBINACA reduced overall mitochondrial motility without altering parameters such as total run length per mobile mitochondrion (51.13 ± 2.55 μm vs. 53.92 ± 6.07 μm in the control, Fig. 5B) and average speed (0.26 ± 0.05 µm/s vs. 0.27 ± 0.04 µm/s in the control, Fig. 5C). No significant differences were observed in the proportion of mitochondria exhibiting anterograde or retrograde movement (Supplementary Information, Figure SI-3).

### Miro1 Levels and Tau Phosphorylation Remained Unaffected Upon Exposure to ADB-FUBINACA

**Fig. 6:**
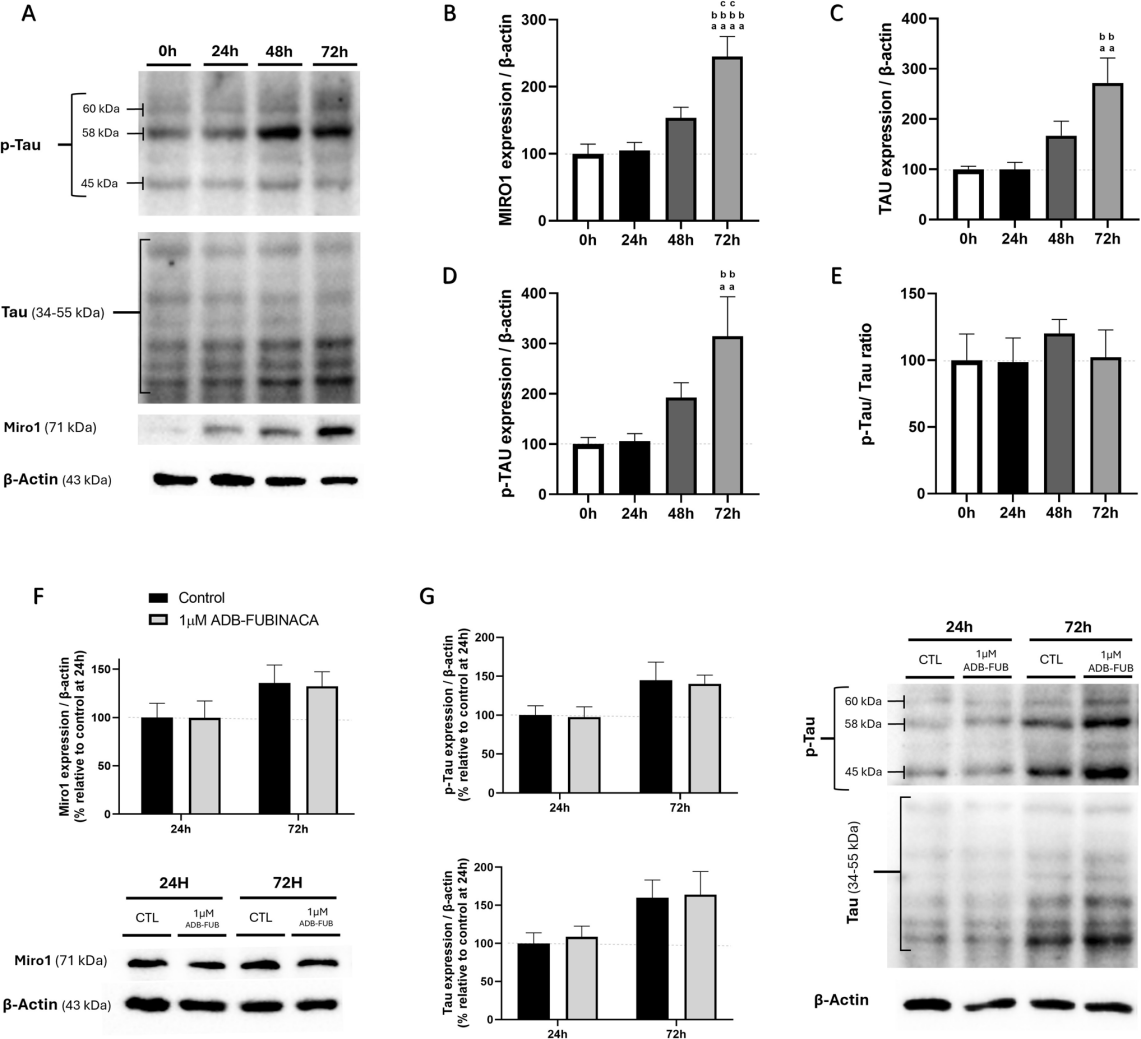
Assessment of mitochondria transport machinery expression levels. (**A**) Representative western blot images. Quantification of (**B**) Miro1 (∼71 kDa), (**C**) Tau (35–55 kDa), (**D**) p-Tau (45, 58 and 60 kDa) and (**E**) p-Tau/Tau ratio over a 72-h period to evaluate their expression profiles during NG108-15 cells differentiation. Evaluation of 1 µM ADB-FUBINACA effects on (**E**) Miro1 and (**F**) p-Tau/Tau levels following 24 and 72 h of exposure. The density of bands was normalized by the amount of β-actin per lane. Each bar represents the mean ± SEM, for at least five independent experiments. ^aa^p < 0.01, ^aaaa^p < 0.0001, compared to vehicle-control at 0 h. ^bb^p < 0.01, ^bbbb^p < 0.0001 compared to vehicle-control at 24 h. ^cc^p < 0.01, compared to vehicle-control at 48 h, using one-way ANOVA followed by Tukey’s post-hoc test

Considering that ADB-FUBINACA alters mitochondrial mobility within neurites, we also ascertained its potential effects on proteins involved in mitochondrial transport. The mitochondrial transport machinery comprises motor proteins that move mitochondria along microtubules, and adaptor proteins, such as Mitochondrial Rho GTPase 1 (Miro1), which tether mitochondria to these motor proteins [45]. We observed that Miro1 levels increased by approximately 2.5-fold at 72 h after the neurodifferentiation of NG108-15 cells was initiated, compared to undifferentiated cells (Fig. 6B).

Moreover, we measured the levels of the microtubule-associated protein Tau and its phosphorylated form (p-Tau), which is known to primarily regulate the assembly and stabilization of microtubules, a process that is crucial for neurite outgrowth and neuronal differentiation. However, excessive phosphorylation of Tau can destabilize microtubules, impairing axonal transport [46]. The six bands detected for Tau likely correspond to the six Tau isoforms (35–56 kDa) that are known to be generated by alternative splicing of the Tau-encoding gene *mapt*, all of which are subject to extensive post-translational modifications, most notably phosphorylation [47].

As represented in Fig. 6C and 6D, both Tau and p-Tau levels significantly increased during the differentiation process, with Tau levels increasing by 2.7-fold and p-Tau by 3.1-fold at 72 h, consistent with the known role of this protein in axonal transport [46]. Despite these increases, the Tau/p-Tau ratio remained consistent throughout the differentiation period (Fig. 6E), indicating that the balance between Tau and its phosphorylated form was maintained during this process. Notably, analysis of these key transport markers showed no changes in cells exposed to 1 µM ADB-FUBINACA (Fig. 6F-6G), indicating that this SC did not impact the adaptor protein levels or microtubule stability.

## Discussion

A proper balance of mitochondrial dynamics is crucial for neuronal differentiation, as it regulates energy production, metabolic signalling, and mitochondrial distribution, which collectively support the growth, maturation, and functionality of developing neurons [2]. Our findings showed that ADB-FUBINACA significantly impacts mitochondrial morphology and dynamics during neurodifferentiation of NG108-15 cells, causing fragmentation of the mitochondrial network in the soma, while mobilizing mitochondria to outgrowing neurites.

During neuronal stem cell differentiation, mitochondria have been reported to undergo significant changes, adopting tubular forms and well-connected networks by the time neurons have formed. This shift towards more elongated mitochondria requires well-regulated expression of mitochondrial fusion and fission proteins [3]. Numerous studies have emphasized the critical role of proteins such as Opa1 [48, 49], Mfn2 [50] and Drp1 [51] in neurodevelopment, while also associating their deficient levels to brain developmental abnormalities. However, the expression patterns of these proteins can vary depending on the specific cellular line. For example, Caglayan et al. reported a 50% increase in total Opa1 mRNA levels during neuronal differentiation of human embryonic stem cells (hESCs) and a 25% increase in neural progenitor cells (NPCs) [52]. Moreover, Soares et al. observed that protein levels of Mfn1 and Mfn2 increased during the differentiation of NSCs from the subventricular zone, while Opa1 levels remained unchanged, and Drp1 levels decreased [53]. In contrast, Vantaggiato et al. observed that neuronal differentiation of embryonal carcinoma P19 cells from mice was accompanied by a progressive increase in Drp1 levels [54]. It is worth noting that, to the best of our knowledge, we herein present first-hand the characterization of the mitochondrial network morphology and the expression profiles of fusion and fission markers in differentiating NG108-15 cells. This characterization contributed to better understand the impact of ADB-FUBINACA on these processes, besides representing an added value for future studies in these cells. Interestingly, we did not observe changes in the morphology of the mitochondrial network between the timepoints evaluated (24 and 72 h). The levels of mitochondrial fusion and fission markers rise concomitantly during neuronal differentiation, implying a synchronized balance in their expression. However, the fission process appears to be constrained by reduced phosphorylation of DRP1 at Ser616, a modification critical for its activation.

In addition, our results indicate that ADB-FUBINACA at biologically relevant concentrations led to substantial alterations in inherent mitochondrial dynamics of differentiating NG108-15, affecting mitochondrial size, shape, and network branching. These changes included a marked shift in the architecture of neuronal mitochondria within the soma, characterized by a fragmented network. Notably, mitochondrial fragmentation is often associated with an imbalance between mitochondrial fission and fusion processes, with excessive fission leading to smaller, isolated mitochondria that are less efficient in ATP production [55]. For instance, previous spatial simulation studies using three-dimensional mitochondrial reconstructions have reported a direct relationship between ATP production rates and mitochondrial structure [56, 57]. Indeed, these morphological alterations may reflect adaptive responses to local variations in energy demand and oxidative phosphorylation activity, particularly the increased ATP turnover required for neurite outgrowth [20]. Herein, we further noted an upregulation of mitochondrial fission proteins (Drp1 and Fis1) along with the suppression of fusion proteins (Opa1, Mfn1, and Mfn2) following ADB-FUBINACA exposure. This aligns with previous studies, where the activation of the CB1 receptor by 20 µM Δ9-THC led to approximately a 50% reduction in fusion protein levels and a doubling of (fission marker) Drp1 in human extravillous trophoblast HTR8/Svneo cells [58] and differentiated human placental BeWo cells [59]. In 2019, Drori et al. demonstrated that intraperitoneal administration of the SC arachidonyl-2'-chloroethylamide (ACEA) to mice (10 mg/kg, i.p.) caused significant mitochondrial fragmentation in renal proximal tubular cells, characterized by higher mitochondrial circularity, reduced perimeter, and diminished interconnectivity [60]. Notably, CB1 receptor knockout mice were protected from these morphological changes and exhibited a hyper-fused mitochondrial network, highlighting the role of CB1 activation in mediating mitochondrial fission/fusion cycles. Likewise, Senese et al. (2024) revealed that CB1 deficiency in mice led to a significant increase in Opa1 levels in skeletal muscle (gastrocnemius), suggesting a shift toward mitochondrial fusion [61]. Remarkably, although ADB-FUBINACA increased fission marker levels in NG108-15 cells, p-Drp1 (Ser616) remains unaffected, suggesting that fission activity was not altered. Consequently, the heightened mitochondrial fragmentation was more likely attributable to diminished fusion rather than increased fission.

Moreover, the reduction in mitochondrial network branching in ADB-FUBINACA-treated cells suggests a reduction in interconnectivity and an increased number of isolated mitochondria. Well-connected mitochondrial networks, characterized by faster fusion and fission dynamics, facilitate efficient distribution of mitochondrial content (e.g. mtDNA, ATP, proteins, ROS) throughout the cell. In contrast, the network fragmentation can delay the spread of critical mitochondrial proteins and molecules, leading to localized energy deficits in specific regions of the soma. Thus, it is reasonable to expect that this disruption may impair the organelles' ability to function as a cohesive unit, ultimately affecting cellular energy distribution and severely impacting neuronal function [62]. Also, changes in mitochondrial morphology regulate mitochondrial function at the level of metabolism and ROS generation, which may act as signalling mechanisms, triggering a cascade of events that lead to the upregulation of genes promoting differentiation while suppressing self-renewal [25]. Thereby, ADB-FUBINACA's modulation of mitochondrial dynamics may be a mechanism that influences mitochondrial-derived metabolic regulators.

In differentiating neurons, efficient mitochondrial transport is crucial, given the need for these organelles to cover long distances to support the neurite terminals [63]. Notably, the upregulation of Miro1 and Tau levels in the control group highlighted the critical role of mitochondrial transport and repositioning during neuronal differentiation. Following the analysis of mitochondria in neurites, we also observed a marked shift in mitochondrial distribution following ADB-FUBINACA exposure. The enhanced mitochondrial presence in neurites can support increased ATP production, necessary for cytoskeletal dynamics [64] and membrane expansion, as well as provide critical signalling molecules [65] and calcium buffering [66], which can further drive neurite outgrowth. ADB-FUBINACA seems to affect mitochondrial mobility by reducing the transport of mitochondria within neurites, possibly attributed to increased mitochondrial anchoring within neurite tracks [67, 68], resulting in a higher proportion of stationary mitochondria, while retaining the movement characteristics of the mobile mitochondria. These findings are consistent with previous studies on the impact of SCs on mitochondrial mobility [69, 70]. For instance, Hebert-Chatelain et al. (2016) demonstrated that 1 µM HU-210 decreased the number of mobile axonal mitochondria in hippocampal neurons via CB1 activation, without affecting mitochondrial velocity, dwelling time, or travel distance [70].

Taken together, our data show that mitochondrial dynamics are not uniformly regulated across the entire neuron, instead they appear to be subject to localized adaptations in neurites that are essential for supporting specific cellular functions. Indeed, several studies have demonstrated that mitochondrial morphology, distribution, and transport can be independently regulated within neurites, highlighting the importance of compartment-specific control mechanisms in facilitating neurite growth and synaptic activity[71–73]. For instance, Virga et al. reported that Ca^2^⁺- and CaMKK2-dependent activation of AMPK locally modulates the fusion–fission balance, thereby regulating mitochondrial morphology at the subcellular level within dendrites [74]. Moreover, these data show that ADB-FUBINACA may preferentially enhance the recruitment of mitochondria within neurites, shifting away from the soma. This prioritization means that while neurites benefit from increased mitochondrial support, which is important for its development and extension, there might be less energy and fewer resources available for the soma. For instance, CD2019, a Retinoic Acid Receptor (RAR)-β agonist, has been shown to promote neurite outgrowth by enhancing the anterograde transport and anchoring of mitochondria in neurites of cultured mouse primary cortical neurons [68]. Notably, the CD2019-mediated effect resulted in mitochondrial membrane depolarization in the soma, implying a redirection of resources to the neurites. Such an imbalance may impact the soma's overall health and functionality, as it relies on a steady supply of energy and mitochondrial support for its own cellular processes and maintenance. Therefore, while ADB-FUBINACA can enhance neurite outgrowth and function, it might affect the soma's performance and overall neuronal health in the long term. To address a potential stage-specificity of the observed responses, it could be interesting to assess the impact of ADB-FUBINACA at later stages of differentiation, as well as on fully differentiated neurons. Although this study did not examine CB1’s role in mitochondrial dynamics, understanding this relationship is also crucial. Our previous work demonstrated that CB1 mediates ADB-FUBINACA–induced effects on neurite outgrowth and mitochondrial function in this cell line [20], supporting a plausible role for CB1R in the responses observed here.

Ultimately, our findings highlight concerns about the safety of SCs, namely ADB-FUBINACA, use during critical neurodevelopmental stages, as our data demonstrate that this SC disrupts dynamic mechanisms of neuronal mitochondria during neuronal cell differentiation, possibly affecting the functional integrity of newly formed neurons. Nonetheless, the mechanisms and potential long-term consequences of these effects need further investigation. Notably, the characterization of key mitochondrial markers during NG108-15 cell differentiation provides valuable insights into mitochondrial remodelling throughout this process, establishing a foundation for future research using this model in related biological contexts. While NG108-15 cells provide a practical model for mechanistic investigation, their tumour-derived origin may limit the generalizability of our findings to human neurons. To enhance translational relevance, future studies should validate these results in more complex *in vitro* systems (e.g. human iPSC-derived neurons), as well as in relevant *in vivo* models.

## Supplementary Information

Below is the link to the electronic supplementary material.

Supplementary file1 (DOCX 68548 KB)

Supplementary file2 (PDF 1740 KB)

Supplementary file3 (GIF 1850 KB)

Supplementary file4 (GIF 25394 KB)

Supplementary file5 (GIF 586 KB)

Supplementary file6 (GIF 6207 KB)

Supplementary file7 (GIF 3296 KB)

Supplementary file8 (GIF 23259 KB)

Supplementary file9 (GIF 781 KB)

Supplementary file10 (GIF 7212 KB)

## Data Availability

Data is provided within the manuscript or supplementary information files.
