# Supplementary material for: The Synthetic Cannabinoid ADB-FUBINACA Disrupts Mitochondrial Morphology and Dynamics during Neuronal Differentiation of NG108-15 Cells
Source: Mol Neurobiol. 2026 Jan 21;63(1):382. doi: 10.1007/s12035-026-05699-x (PMC12823749; doi:10.1007/s12035-026-05699-x)
Supplement: Supplementary file 1 — Supplementary file1 (DOCX 68548 KB) [file 12035_2026_5699_MOESM1_ESM.docx]

**The synthetic cannabinoid ADB-FUBINACA disrupts mitochondrial morphology and dynamics during neuronal differentiation of NG108-15 cells**

**- Supplementary Information -**

Rui Filipe Malheiro^1,2^, Ana Catarina Costa^3^, Helena Carmo^1,2^, Félix Carvalho^1,2,*^, João Pedro Silva^1,2,*^

^1^ Applied Molecular Biosciences Unit (UCIBIO), Laboratory of Toxicology, Department of Biological Sciences, Faculty of Pharmacy, Rua Jorge de Viterbo Ferreira 228, University of Porto, 4050-313 Porto, Portugal.

^2^ Associate Laboratory Institute for Health and Bioeconomy (i4HB), Faculty of Pharmacy, Rua Jorge de Viterbo Ferreira 228, University of Porto, 4050-313, Porto, Portugal.

^3^ Nerve Regeneration Group, Instituto de Biologia Molecular e Celular (IBMC), Instituto de Investigação e Inovação em Saúde (i3S), University of Porto, 4200-135 Porto, Portugal

*Co-senior and corresponding authors:

João P. Silva, Félix Carvalho,

Associate Laboratory i4HB - Institute for Health and Bioeconomy, UCIBIO, Laboratory of Toxicology, Department of Biological Sciences, Faculty of Pharmacy, University of Porto, 4050-313, Porto, Portugal

E-mails: jpmsilva@ff.up.pt, felixdc@ff.up.pt

Tel: +351 220428600


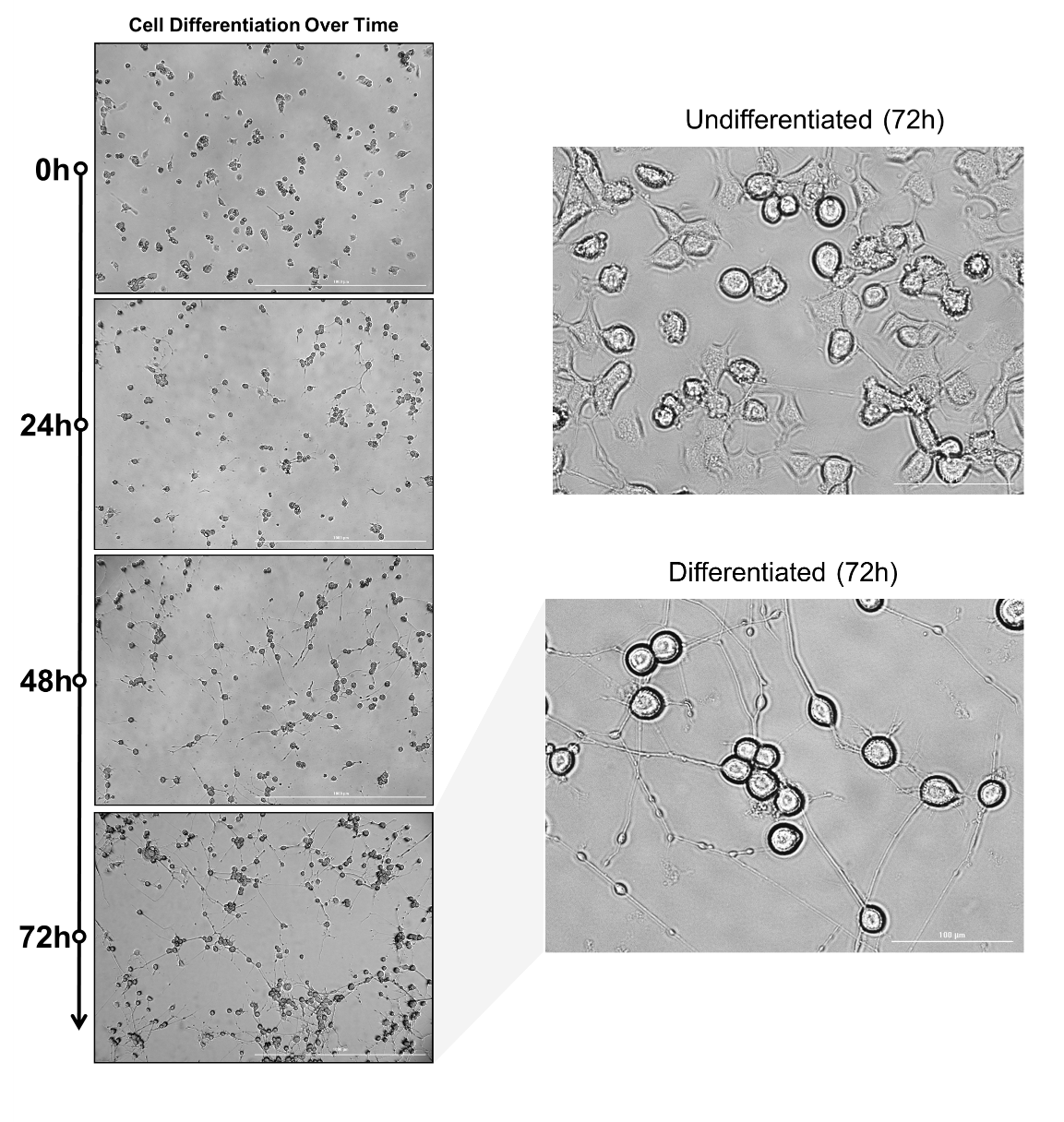


**Figure SI-1 - Representative bright-field microscopy images of NG108-15 cells captured over 72 h of differentiation**, induced in serum-starved medium supplemented with forskolin and retinoic acid.


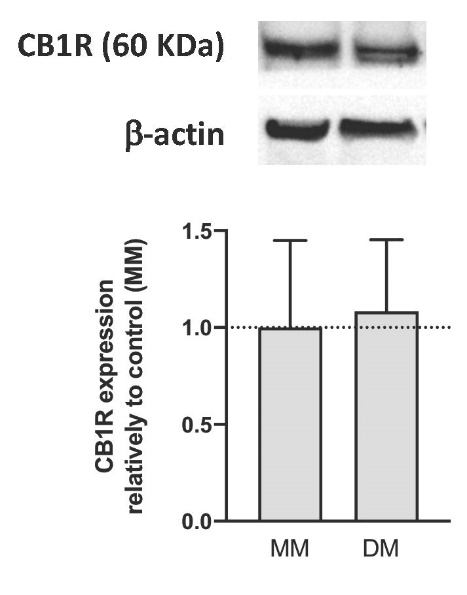


**Figure SI-2. The expression of the CB1 receptor (60 kDa) in NG108-15 cells was assessed by Western blot after 72 hours of differentiation, compared to non-differentiated cells.** Representative protein bands and graphical representations of band intensities, were expressed relatively to non-differentiated cells. Their expression was normalized by the amount of β-actin per lane. Each bar represents the mean ± SD, for three independent experiments. MM: Maintenance Medium, DM: Differentiation Medium.


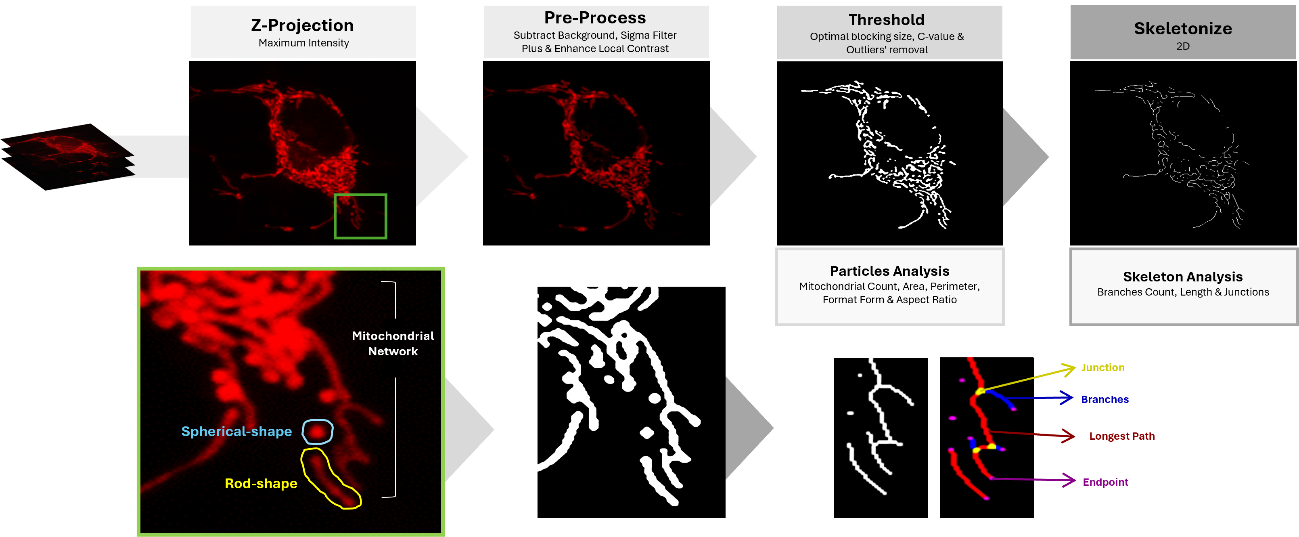


**Figure SI-3 - Summary pipeline for 2-dimensional (2D) analysis of mitochondrial morphology using “Mitochondria Analyzer” plugin in ImageJ/Fiji.** Cells with clear signal-to-noise contrast in both the soma and neurites were selected to ensure high-quality results, and images were captured through multiple focal planes (Z-stacks). A Z-projection using maximum intensity was then applied to merge these slices into a single 2D image. Subsequently, several image preprocessing techniques were employed to enhance quality, including “Background Subtraction”, “Local Contrast Enhancement”, and “Sigma Filtering”. Optimized parameters, including block size and C values, were applied to threshold images using the weighted mean method. The resulting binary images were then refined through post-processing with the “Despeckle” and “Remove Outliers” commands. Following segmentation, the "Analyze Particles" command was used to extract key morphological features and metrics from the mitochondrial objects. These features included mitochondrial count, area, perimeter, form factor, and aspect ratio. Additionally, the thresholded particles were converted into topological skeletons using the “Skeletonize (2D/3D)” function. This process allowed for the assessment of mitochondrial network connectivity by analysing and quantifying the number of branches, branch lengths, and branch junctions within the network.

A

B

**Figure SI-4 – Percentage of mitochondria exhibiting (A) anterograde and (B) retrograde movement within neurites.** Mitochondrial tracking and analysis were performed using the MTrackJ plugin.


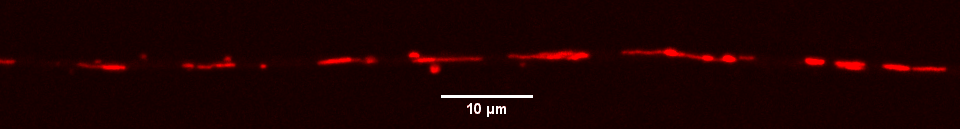
**Control (**20x Real-time**)**


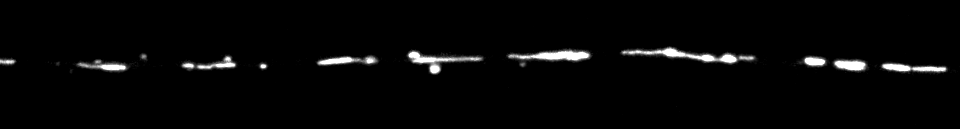


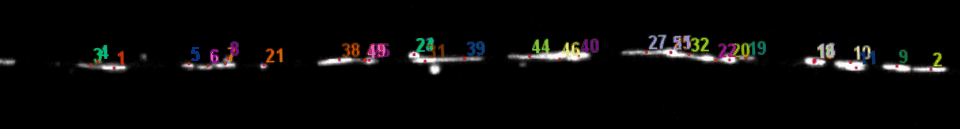


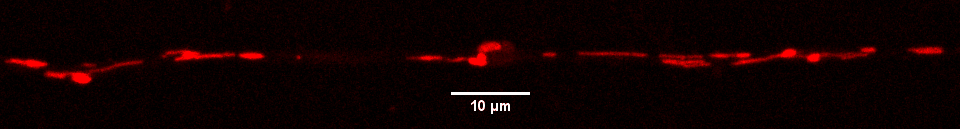
**1μM ADB-FUBINACA (**20x Real-time**)**


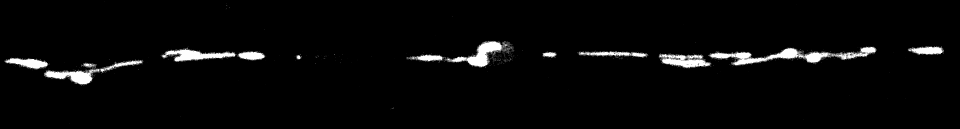


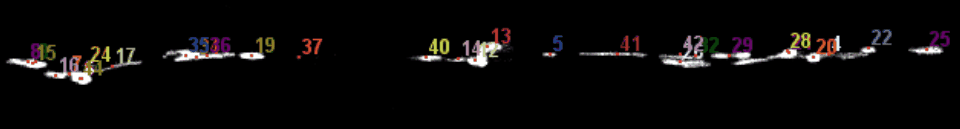


**Figure SI-4 – Evaluation of mitochondrial mobility in NG108-15 cell neurites labeled with PKmito RED.** Tracking mitochondrial movement along 100 μm neurite segments over 10 minutes, analyzed using the MTrackJ plugin.Time-lapses displayed at 20 times the real-time speed.
