# Supplementary material for: The Synthetic Cannabinoid ADB-FUBINACA Disrupts Mitochondrial Morphology and Dynamics during Neuronal Differentiation of NG108-15 Cells
Source: Mol Neurobiol. 2026 Jan 21;63(1):382. doi: 10.1007/s12035-026-05699-x (PMC12823749; doi:10.1007/s12035-026-05699-x)
Supplement: Supplementary file 2 — Supplementary file2 (PDF 1740 KB) [file 12035_2026_5699_MOESM2_ESM.pdf]

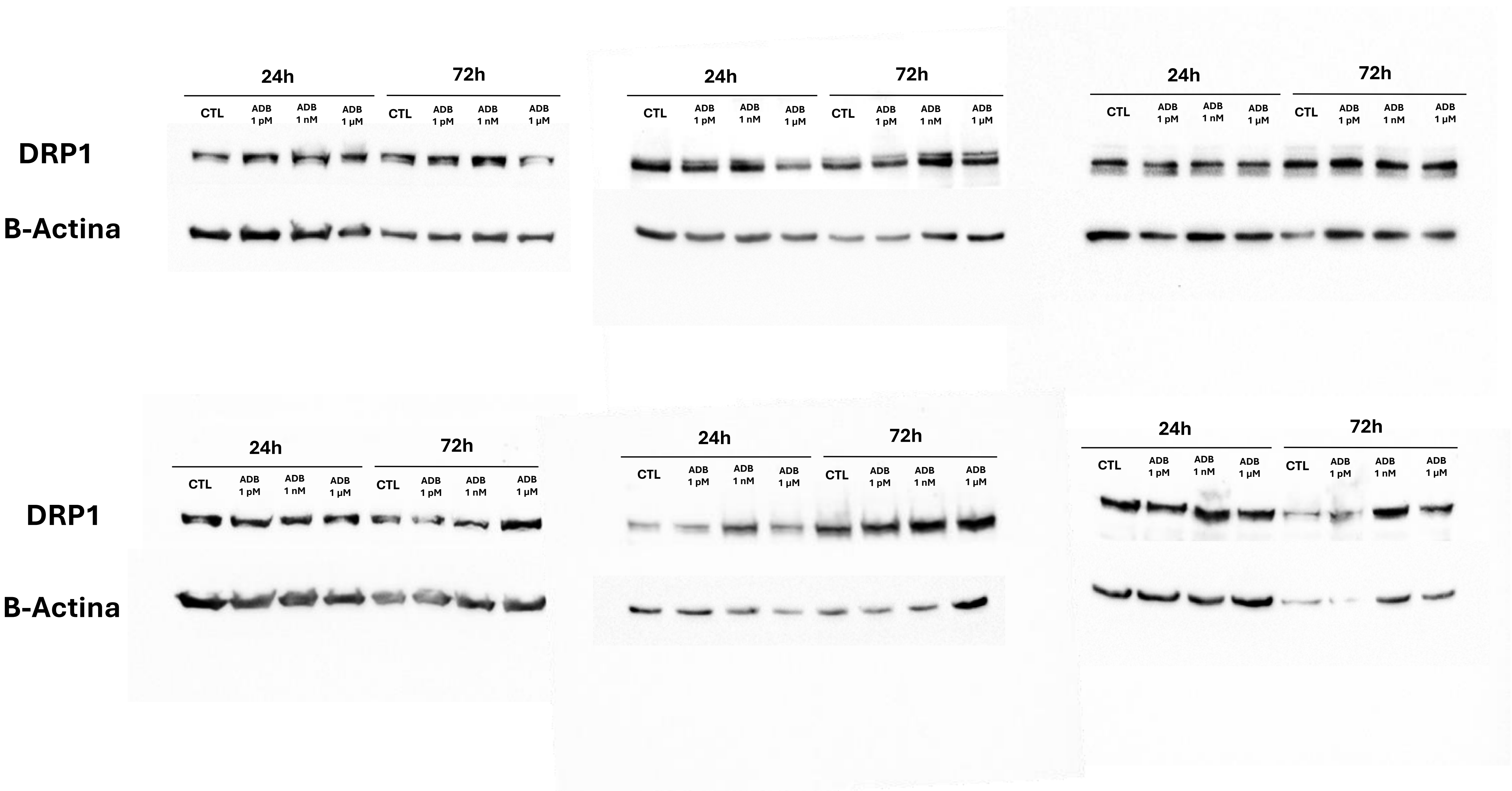
DRP1
B-Actina
24h
72h
CTL
ADB 1 pM
ADB 1 nM
ADB 1 μM
DRP1
B-Actina

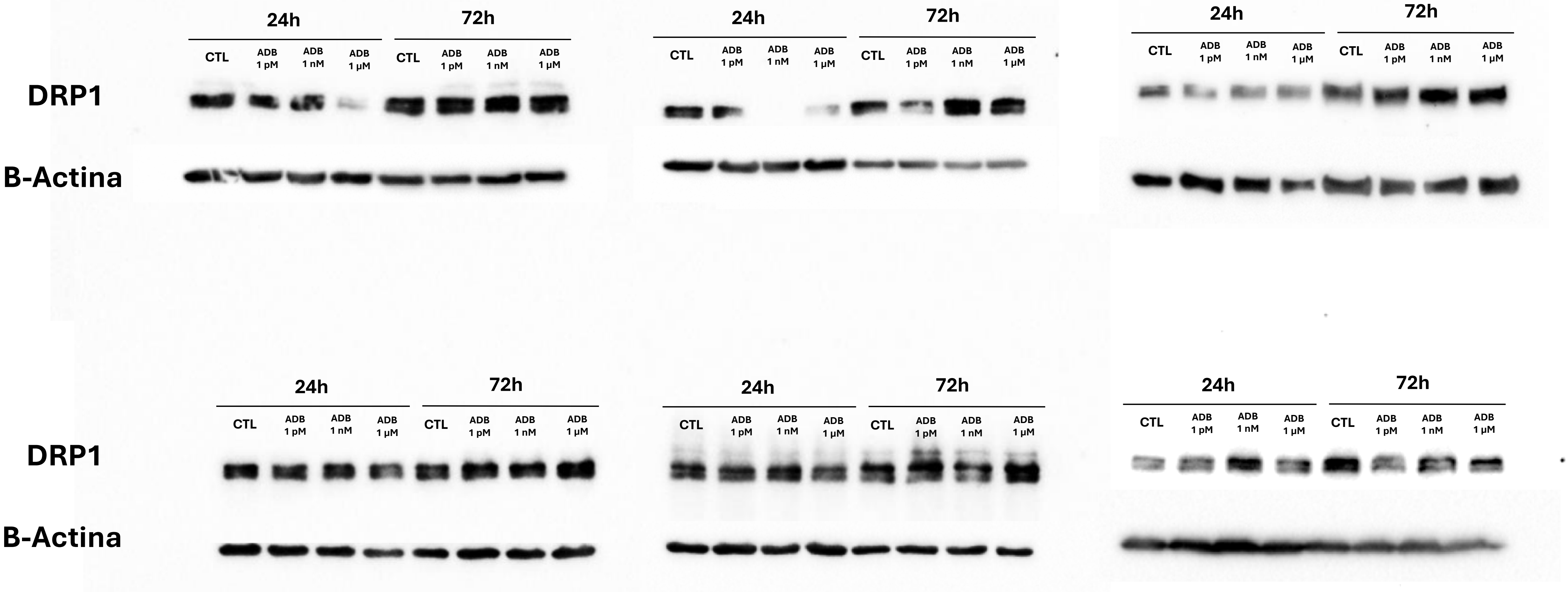

DRP1
B-Actina
24h
72h
CTL
ADB 1 pM
ADB 1 nM
ADB 1 µM

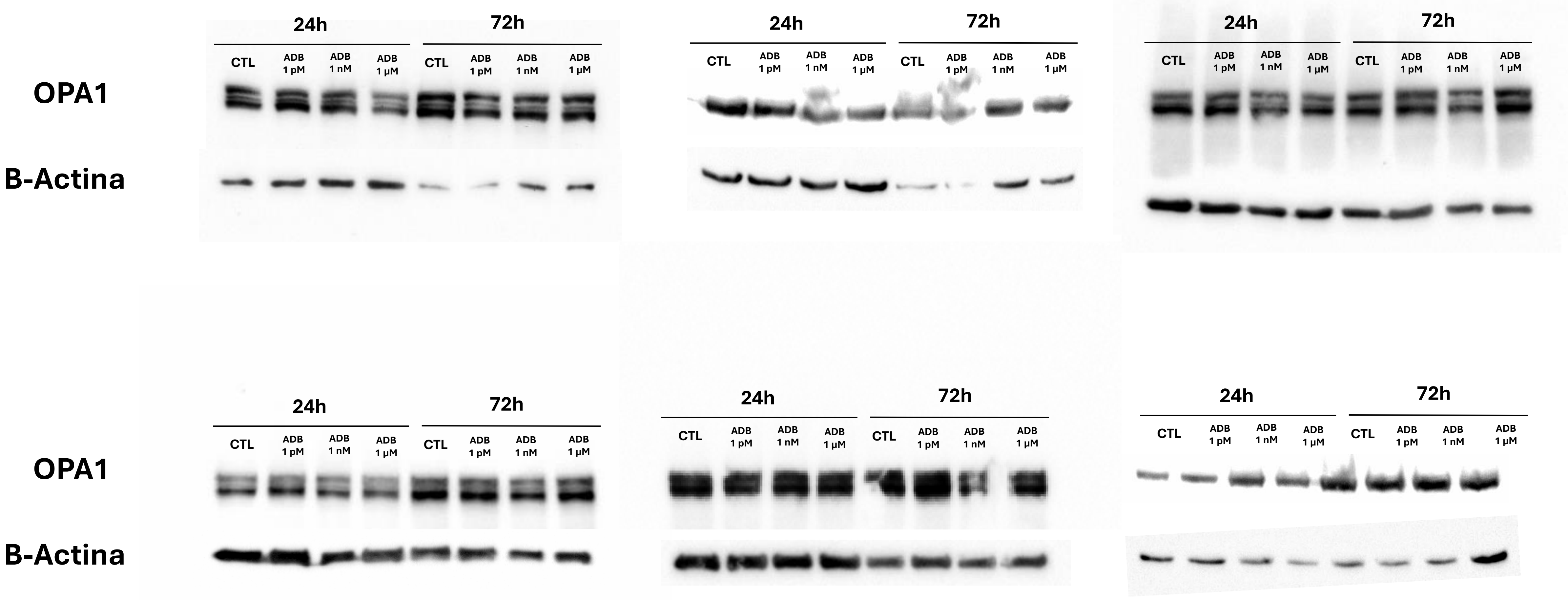

OPA1
B-Actina
24h
72h
CTL
ADB 1 pM
ADB 1 nM
ADB 1 µM
CTL
ADB 1 pM
ADB 1 nM
ADB 1 µM
24h
72h
CTL
ADB 1 pM
ADB 1 nM
ADB 1 µM
CTL
ADB 1 pM
ADB 1 nM
ADB 1 µM
24h
72h
CTL
ADB 1 pM
ADB 1 nM
ADB 1 µM
CTL
ADB 1 pM
ADB 1 nM
ADB 1 µM
OPA1
B-Actina
24h
72h
CTL
ADB 1 pM
ADB 1 nM
ADB 1 µM
CTL
ADB 1 pM
ADB 1 nM
ADB 1 µM
24h
72h
CTL
ADB 1 pM
ADB 1 nM
ADB 1 µM
CTL
ADB 1 pM
ADB 1 nM
ADB 1 µM
24h
72h
CTL
ADB 1 pM
ADB 1 nM
ADB 1 µM
CTL
ADB 1 pM
ADB 1 nM
ADB 1 µM

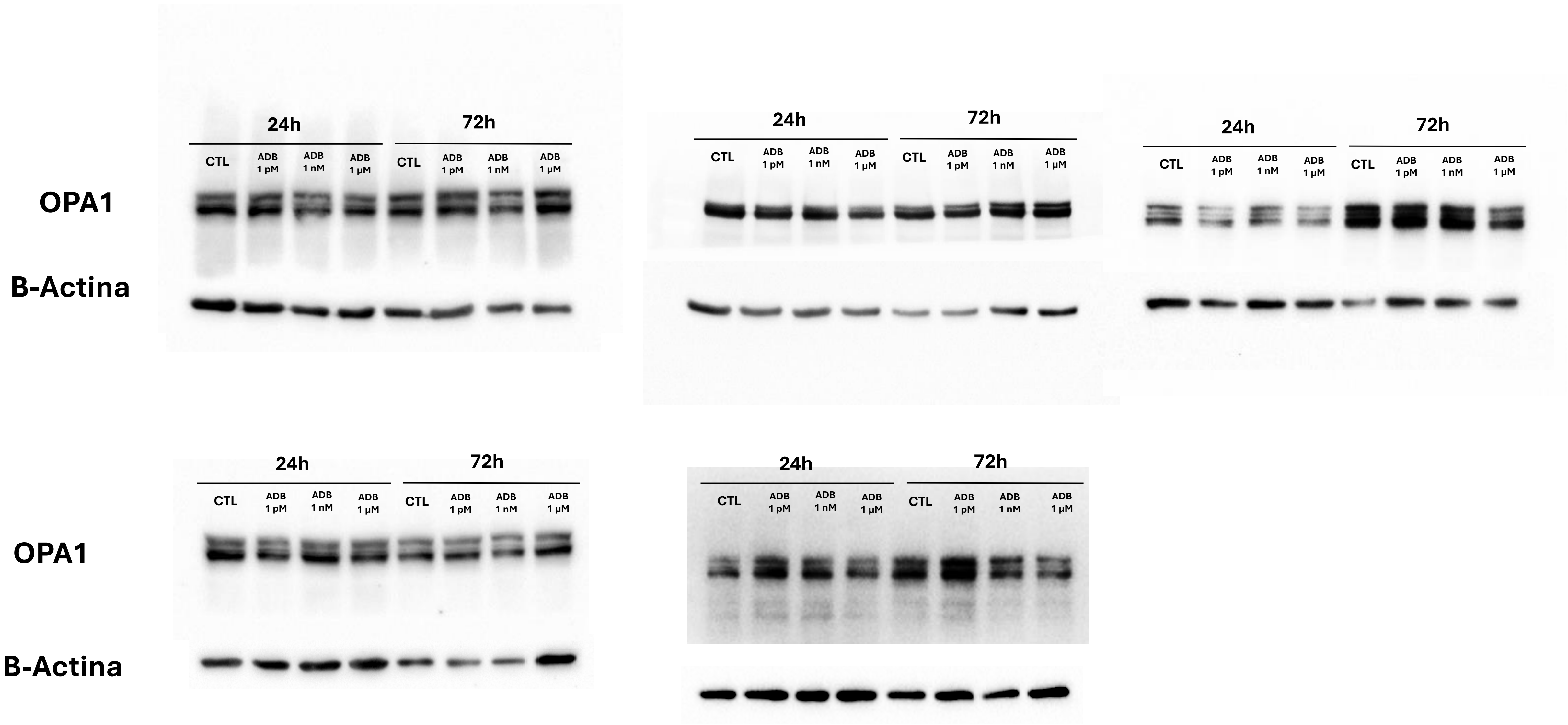
24h
72h
CTL
ADB 1 pM
ADB 1 nM
ADB 1 µM
CTL
ADB 1 pM
ADB 1 nM
ADB 1 µM
OPA1
B-Actina
24h
72h
CTL
ADB 1 pM
ADB 1 nM
ADB 1 µM
CTL
ADB 1 pM
ADB 1 nM
ADB 1 µM
24h
72h
CTL
ADB 1 pM
ADB 1 nM
ADB 1 µM
CTL
ADB 1 pM
ADB 1 nM
ADB 1 µM
24h
72h
CTL
ADB 1 pM
ADB 1 nM
ADB 1 µM
CTL
ADB 1 pM
ADB 1 nM
ADB 1 µM
OPA1
B-Actina
24h
72h
CTL
ADB 1 pM
ADB 1 nM
ADB 1 µM
CTL
ADB 1 pM
ADB 1 nM
ADB 1 µM

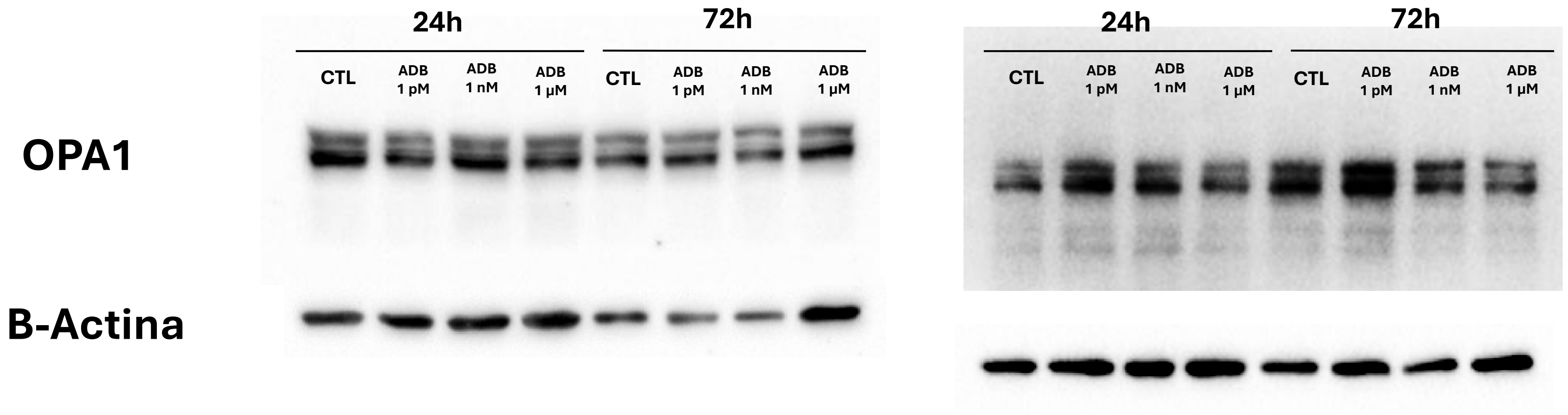
24h
72h
CTL
ADB 1 pM
ADB 1 nM
ADB 1 μM
CTL
ADB 1 pM
ADB 1 nM
ADB 1 μM
OPA1
B-Actina
24h
72h
CTL
ADB 1 pM
ADB 1 nM
ADB 1 μM
CTL
ADB 1 pM
ADB 1 nM
ADB 1 μM

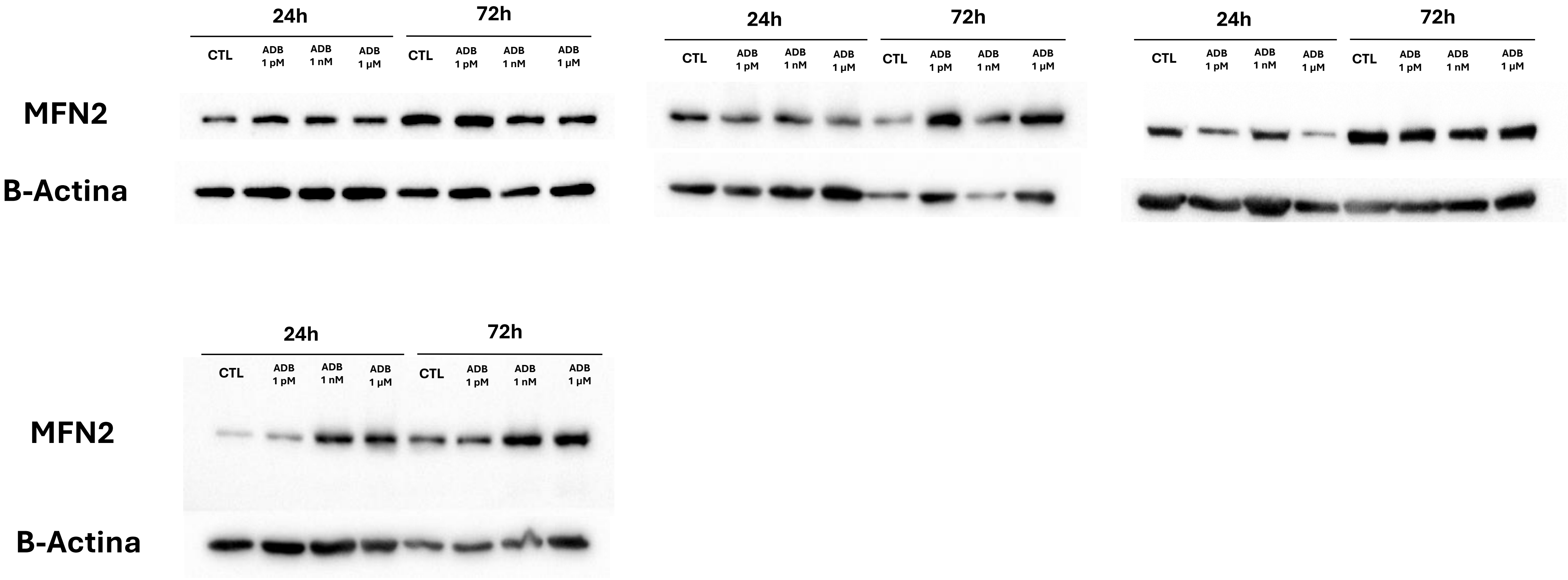
24h
72h
CTL
ADB 1 pM
ADB 1 nM
ADB 1 µM
CTL
ADB 1 pM
ADB 1 nM
ADB 1 µM
MFN2
B-Actina
24h
72h
CTL
ADB 1 pM
ADB 1 nM
ADB 1 µM
CTL
ADB 1 pM
ADB 1 nM
ADB 1 µM
24h
72h
CTL
ADB 1 pM
ADB 1 nM
ADB 1 µM
CTL
ADB 1 pM
ADB 1 nM
ADB 1 µM
24h
72h
CTL
ADB 1 pM
ADB 1 nM
ADB 1 µM
CTL
ADB 1 pM
ADB 1 nM
ADB 1 µM
MFN2
B-Actina

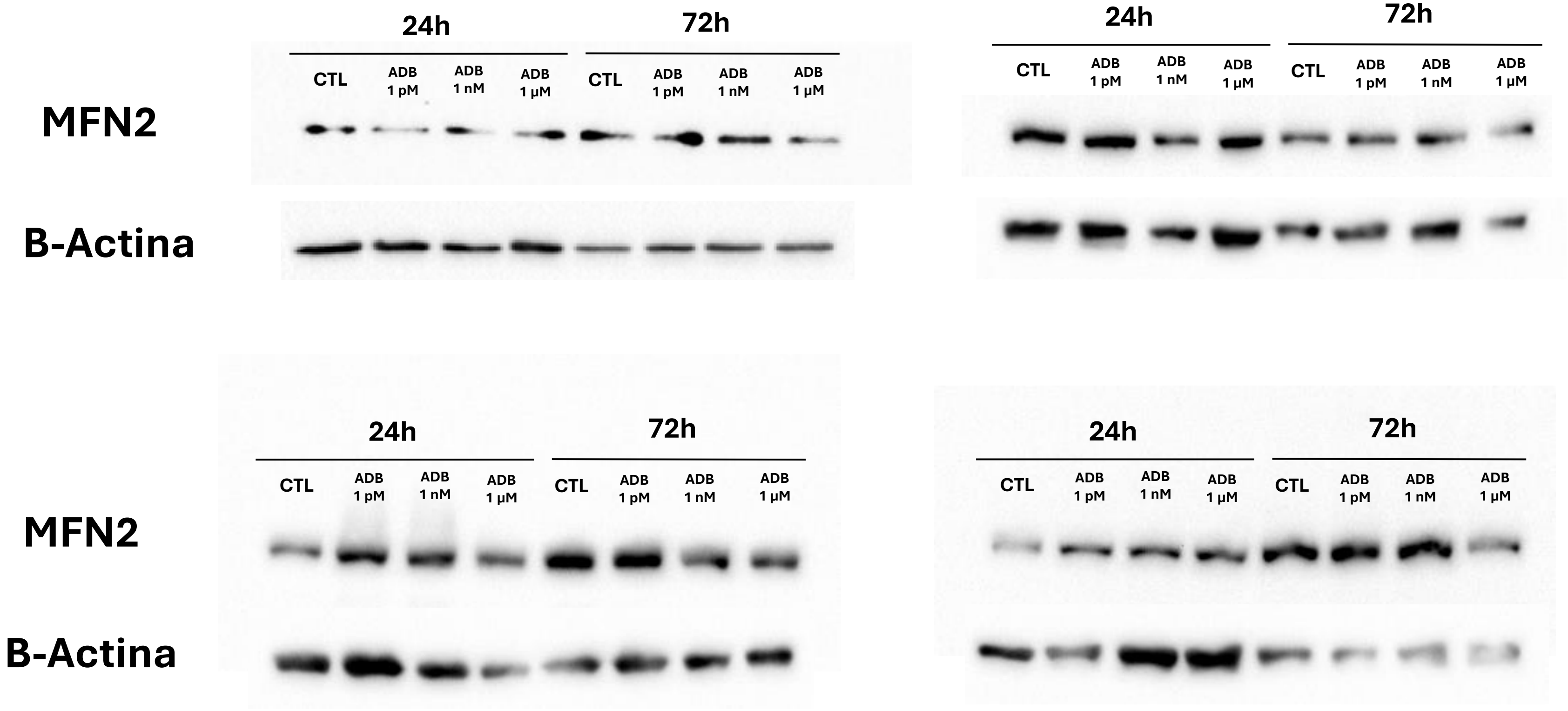

MFN2
B-Actina
24h
72h
CTL
ADB 1 pM
ADB 1 nM
ADB 1 μM
CTL
ADB 1 pM
ADB 1 nM
ADB 1 μM
24h
72h
CTL
ADB 1 pM
ADB 1 nM
ADB 1 μM
CTL
ADB 1 pM
ADB 1 nM
ADB 1 μM
MFN2
B-Actina
24h
72h
CTL
ADB 1 pM
ADB 1 nM
ADB 1 μM
CTL
ADB 1 pM
ADB 1 nM
ADB 1 μM
24h
72h
CTL
ADB 1 pM
ADB 1 nM
ADB 1 μM
CTL
ADB 1 pM
ADB 1 nM
ADB 1 μM

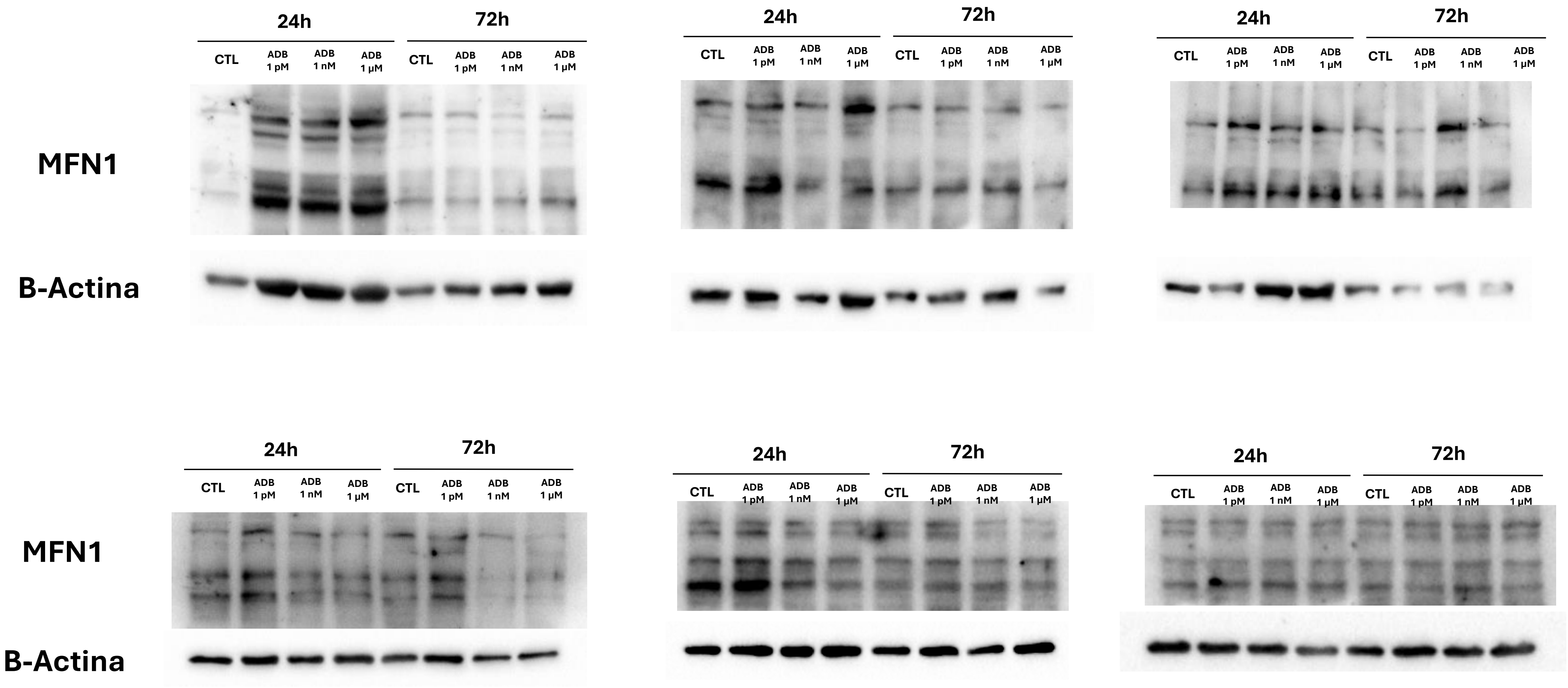
24h
72h
CTL
ADB 1 pM
ADB 1 nM
ADB 1 μM
MFN1
B-Actina

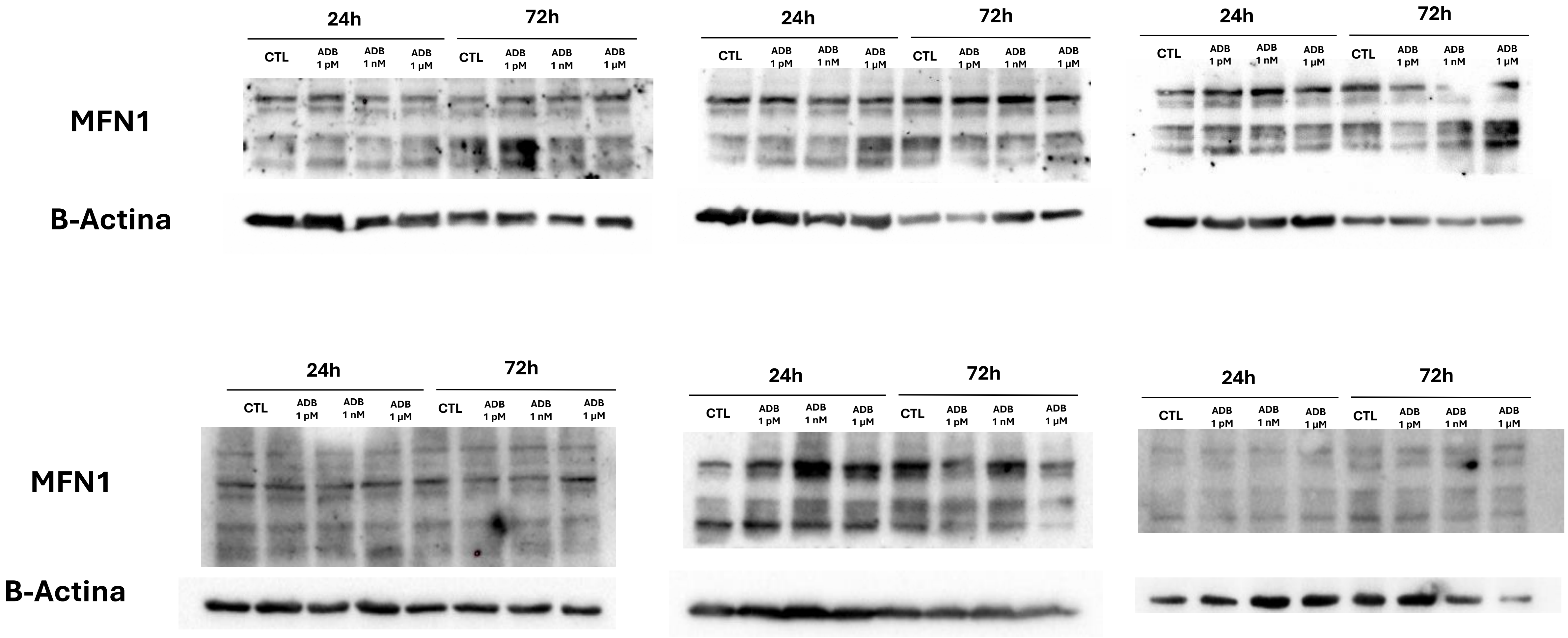
MFN1
B-Actina
24h
72h
CTL
ADB 1 pM
ADB 1 nM
ADB 1 μM
CTL
ADB 1 pM
ADB 1 nM
ADB 1 μM
24h
72h
CTL
ADB 1 pM
ADB 1 nM
ADB 1 μM
CTL
ADB 1 pM
ADB 1 nM
ADB 1 μM
24h
72h
CTL
ADB 1 pM
ADB 1 nM
ADB 1 μM
CTL
ADB 1 pM
ADB 1 nM
ADB 1 μM
MFN1
B-Actina
24h
72h
CTL
ADB 1 pM
ADB 1 nM
ADB 1 μM
CTL
ADB 1 pM
ADB 1 nM
ADB 1 μM
24h
72h
CTL
ADB 1 pM
ADB 1 nM
ADB 1 μM
CTL
ADB 1 pM
ADB 1 nM
ADB 1 μM
24h
72h
CTL
ADB 1 pM
ADB 1 nM
ADB 1 μM
CTL
ADB 1 pM
ADB 1 nM
ADB 1 μM

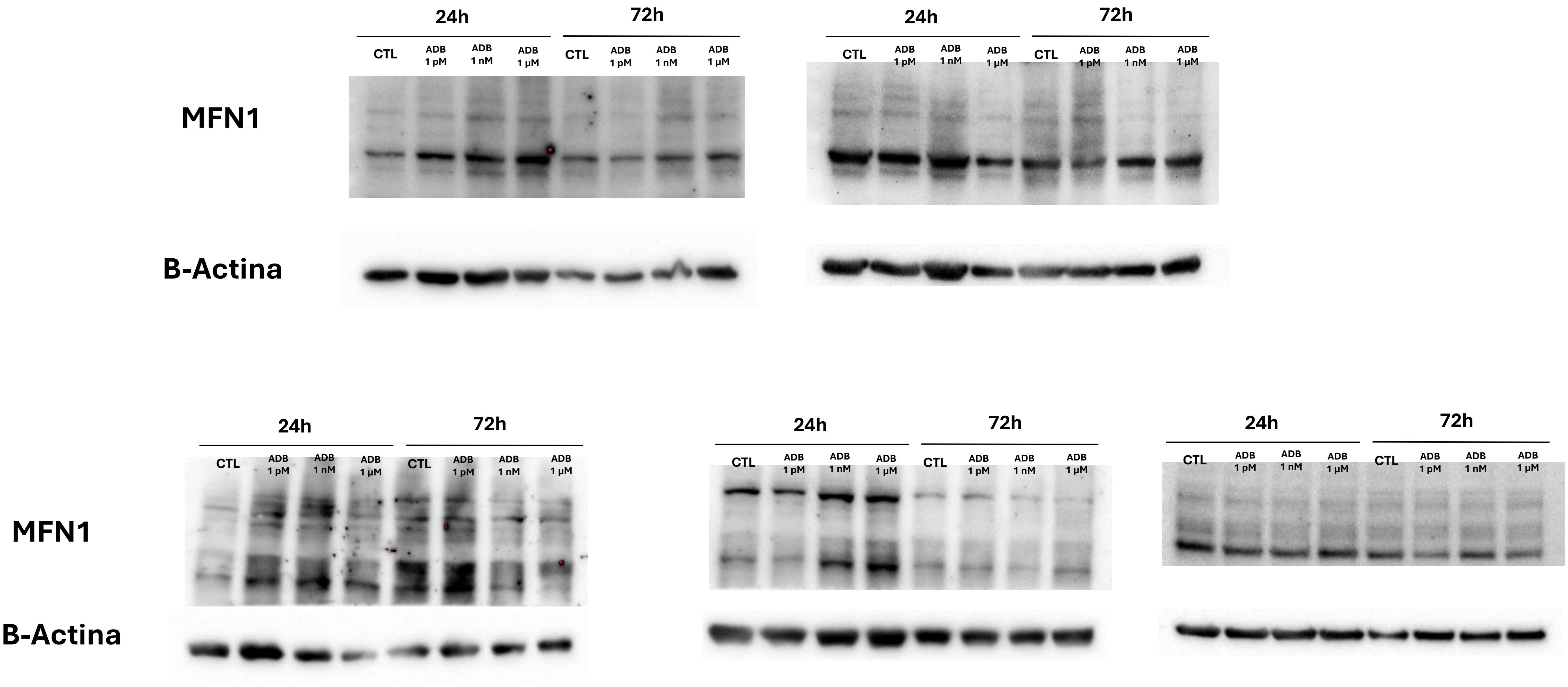
MFN1
B-Actina
24h
72h
CTL
ADB 1 pM
ADB 1 nM
ADB 1 μM
CTL
ADB 1 pM
ADB 1 nM
ADB 1 μM
24h
72h
CTL
ADB 1 pM
ADB 1 nM
ADB 1 μM
CTL
ADB 1 pM
ADB 1 nM
ADB 1 μM
MFN1
B-Actina
24h
72h
CTL
ADB 1 pM
ADB 1 nM
ADB 1 μM
CTL
ADB 1 pM
ADB 1 nM
ADB 1 μM
24h
72h
CTL
ADB 1 pM
ADB 1 nM
ADB 1 μM
CTL
ADB 1 pM
ADB 1 nM
ADB 1 μM
24h
72h
CTL
ADB 1 pM
ADB 1 nM
ADB 1 μM
CTL
ADB 1 pM
ADB 1 nM
ADB 1 μM

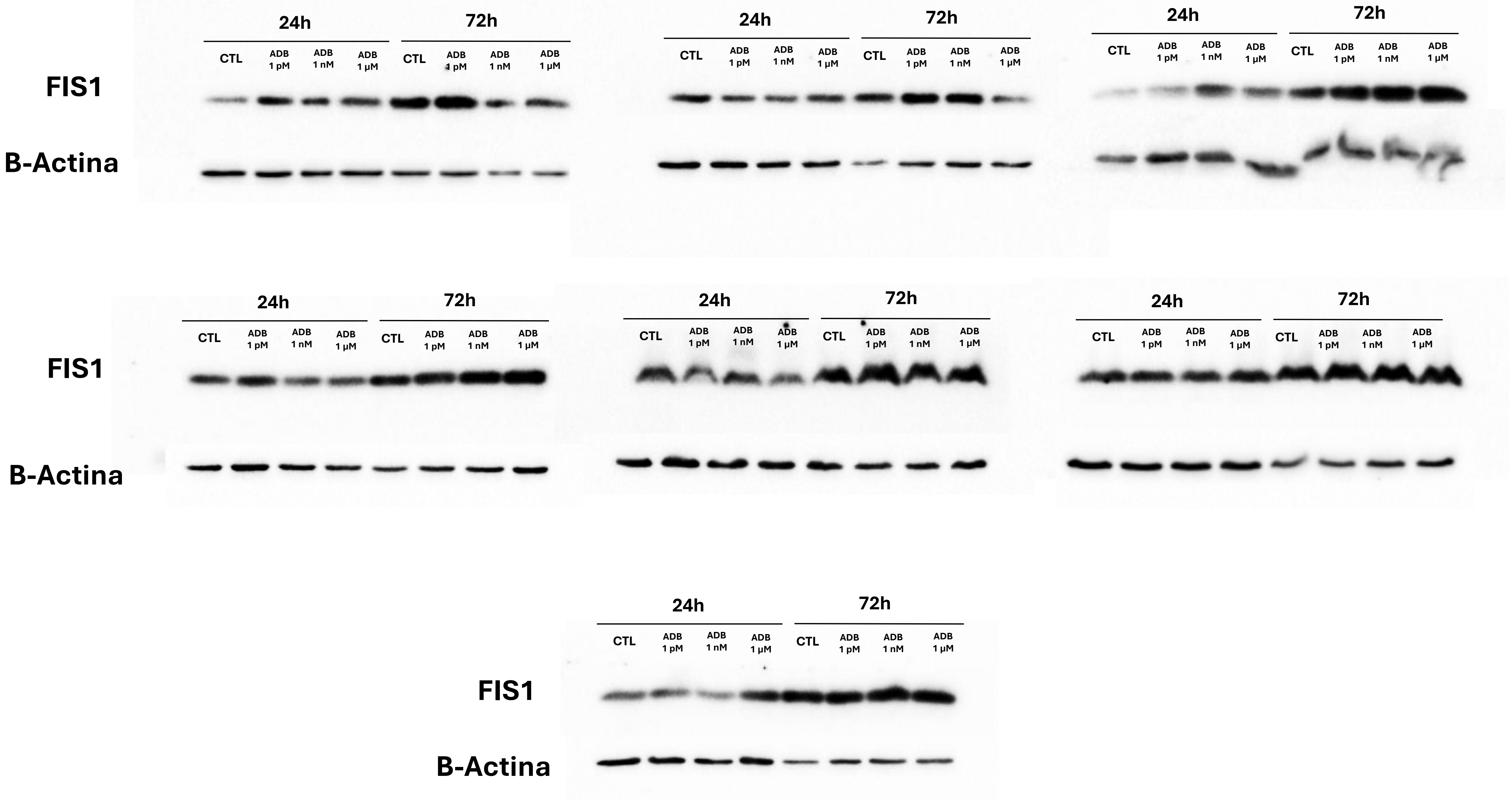
24h
72h
CTL
ADB 1 pM
ADB 1 nM
ADB 1 µM
CTL
ADB 1 pM
ADB 1 nM
ADB 1 µM
FIS1
B-Actina
24h
72h
CTL
ADB 1 pM
ADB 1 nM
ADB 1 µM
CTL
ADB 1 pM
ADB 1 nM
ADB 1 µM
24h
72h
CTL
ADB 1 pM
ADB 1 nM
ADB 1 µM
CTL
ADB 1 pM
ADB 1 nM
ADB 1 µM
24h
72h
CTL
ADB 1 pM
ADB 1 nM
ADB 1 µM
CTL
ADB 1 pM
ADB 1 nM
ADB 1 µM
FIS1
B-Actina
24h
72h
CTL
ADB 1 pM
ADB 1 nM
ADB 1 µM
CTL
ADB 1 pM
ADB 1 nM
ADB 1 µM
24h
72h
CTL
ADB 1 pM
ADB 1 nM
ADB 1 µM
CTL
ADB 1 pM
ADB 1 nM
ADB 1 µM
24h
72h
CTL
ADB 1 pM
ADB 1 nM
ADB 1 µM
CTL
ADB 1 pM
ADB 1 nM
ADB 1 µM
FIS1
B-Actina

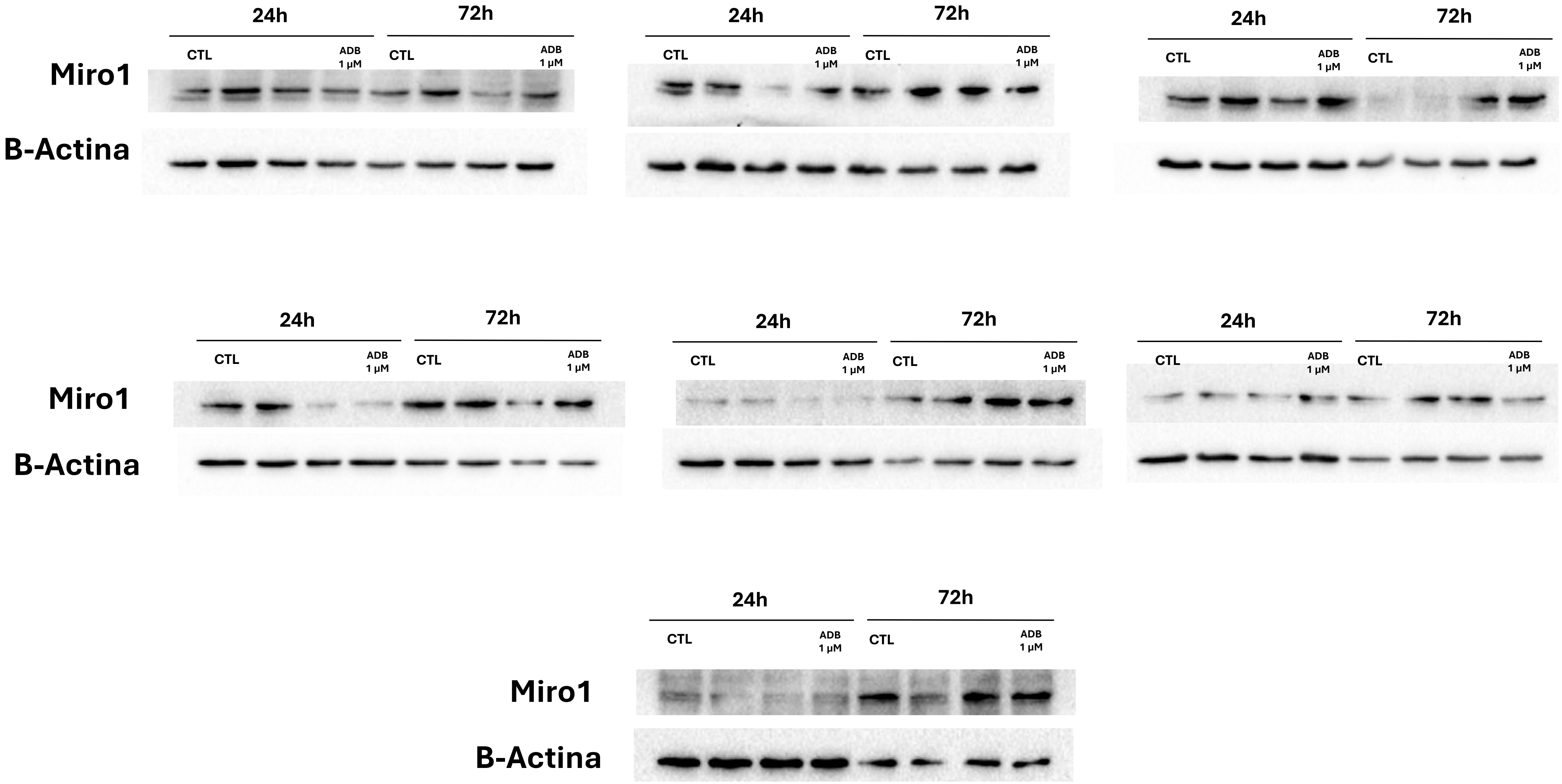
24h
72h
CTL
ADB
1 μM
CTL
ADB
1 μM
Miro1
B-Actina
24h
72h
CTL
ADB
1 μM
CTL
ADB
1 μM
24h
72h
CTL
ADB
1 μM
CTL
ADB
1 μM
24h
72h
CTL
ADB
1 μM
CTL
ADB
1 μM
Miro1
B-Actina
24h
72h
CTL
ADB
1 μM
CTL
ADB
1 μM
24h
72h
CTL
ADB
1 μM
CTL
ADB
1 μM
24h
72h
CTL
ADB
1 μM
CTL
ADB
1 μM
Miro1
B-Actina

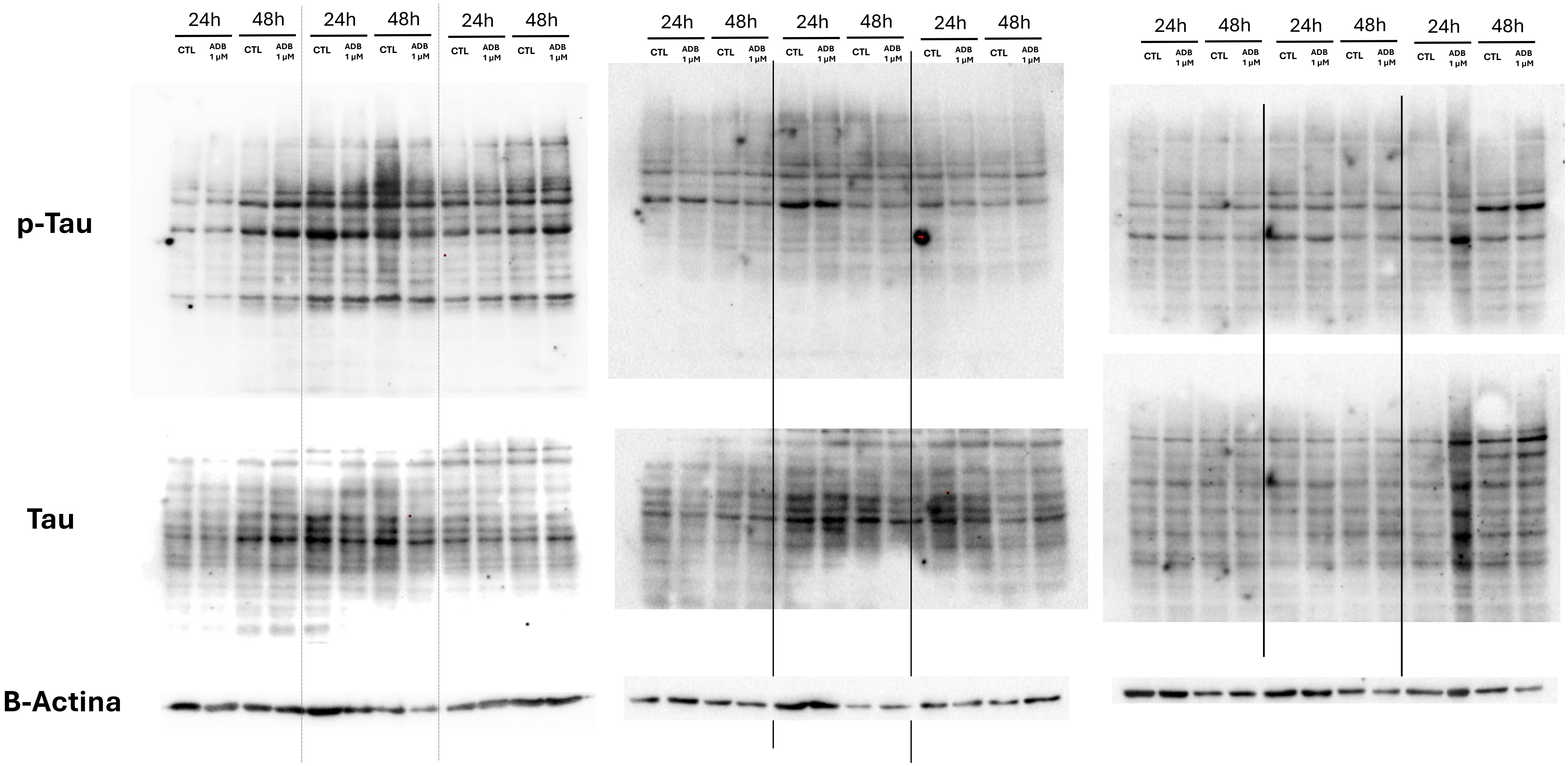

p-Tau
Tau
B-Actina
24h
48h
CTL
ADB 1 µM

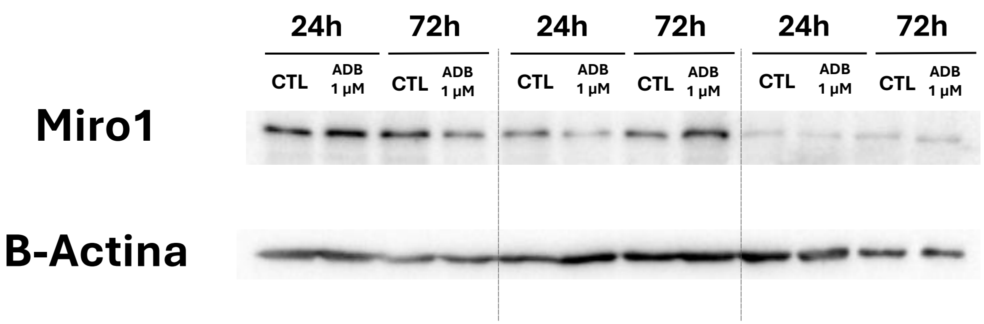
24h
72h
24h
72h
24h
72h
CTL
ADB 1 μM
CTL
ADB 1 μM
CTL
ADB 1 μM
CTL
ADB 1 μM
CTL
ADB 1 μM
CTL
ADB 1 μM
Miro1
B-Actina

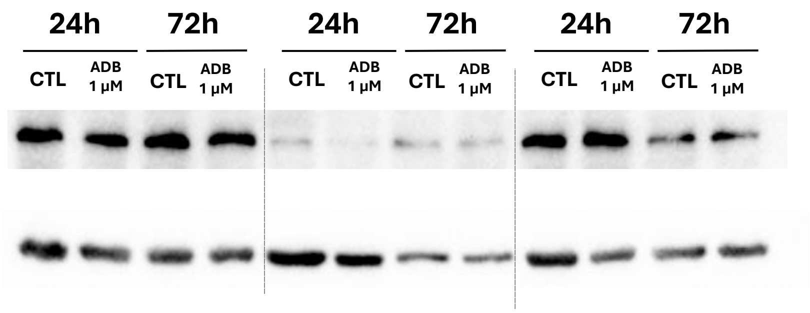
24h
72h
24h
72h
24h
72h
CTL
ADB 1 μM
CTL
ADB 1 μM
CTL
ADB 1 μM
CTL
ADB 1 μM
CTL
ADB 1 μM
CTL
ADB 1 μM

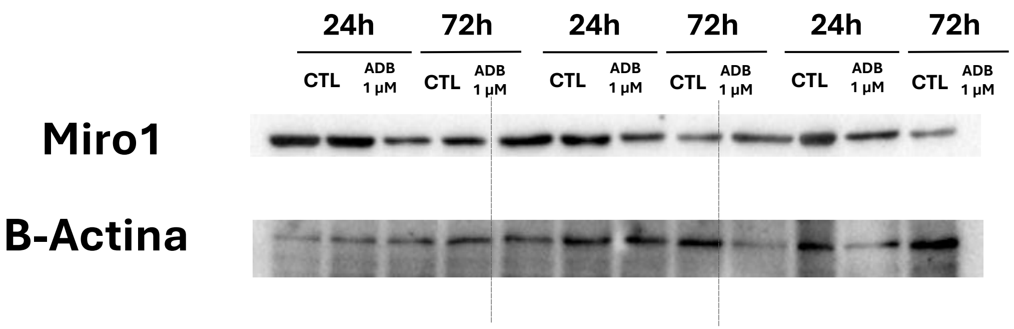
24h
72h
24h
72h
24h
72h
CTL
ADB 1 μM
CTL
ADB 1 μM
CTL
ADB 1 μM
CTL
ADB 1 μM
CTL
ADB 1 μM
CTL
ADB 1 μM
Miro1
B-Actina

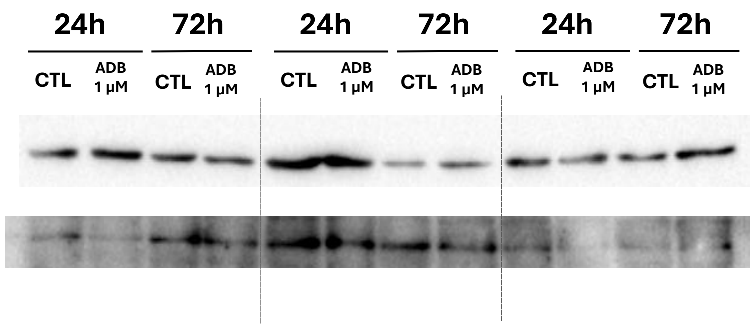
24h
72h
24h
72h
24h
72h
CTL
ADB 1 μM
CTL
ADB 1 μM
CTL
ADB 1 μM
CTL
ADB 1 μM
CTL
ADB 1 μM
CTL
ADB 1 μM

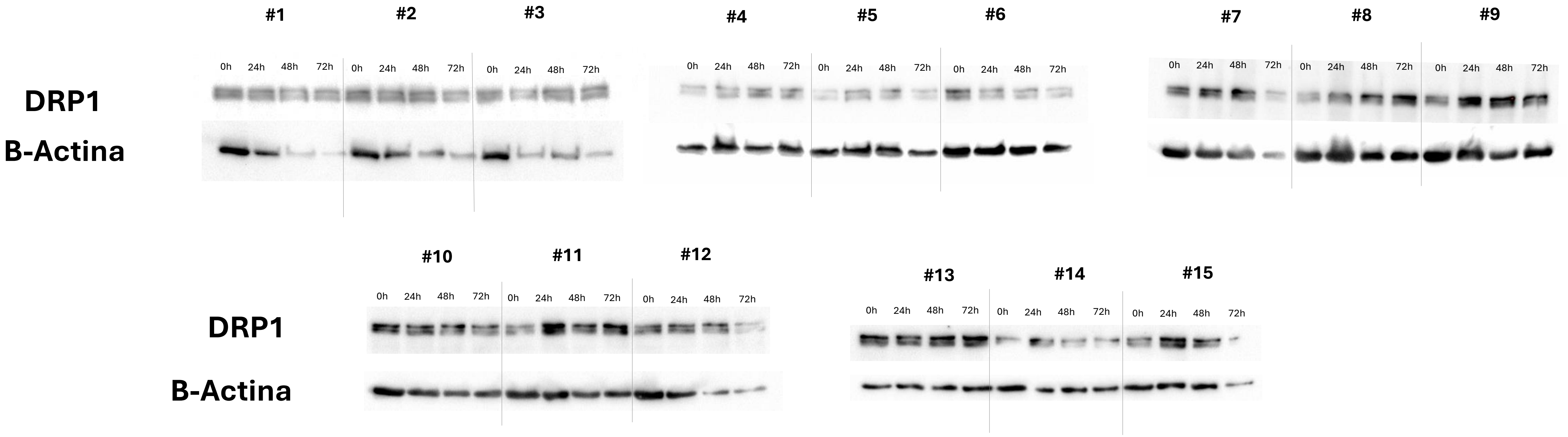
#1
#2
#3
#4
#5
#6
#7
#8
#9
0h 24h 48h 72h 0h 24h 48h 72h 0h 24h 48h 72h
0h 24h 48h 72h 0h 24h 48h 72h 0h 24h 48h 72h
0h 24h 48h 72h 0h 24h 48h 72h 0h 24h 48h 72h
DRP1
B-Actina
#10
#11
#12
#13
#14
#15
0h 24h 48h 72h 0h 24h 48h 72h 0h 24h 48h 72h
0h 24h 48h 72h 0h 24h 48h 72h 0h 24h 48h 72h
DRP1
B-Actina

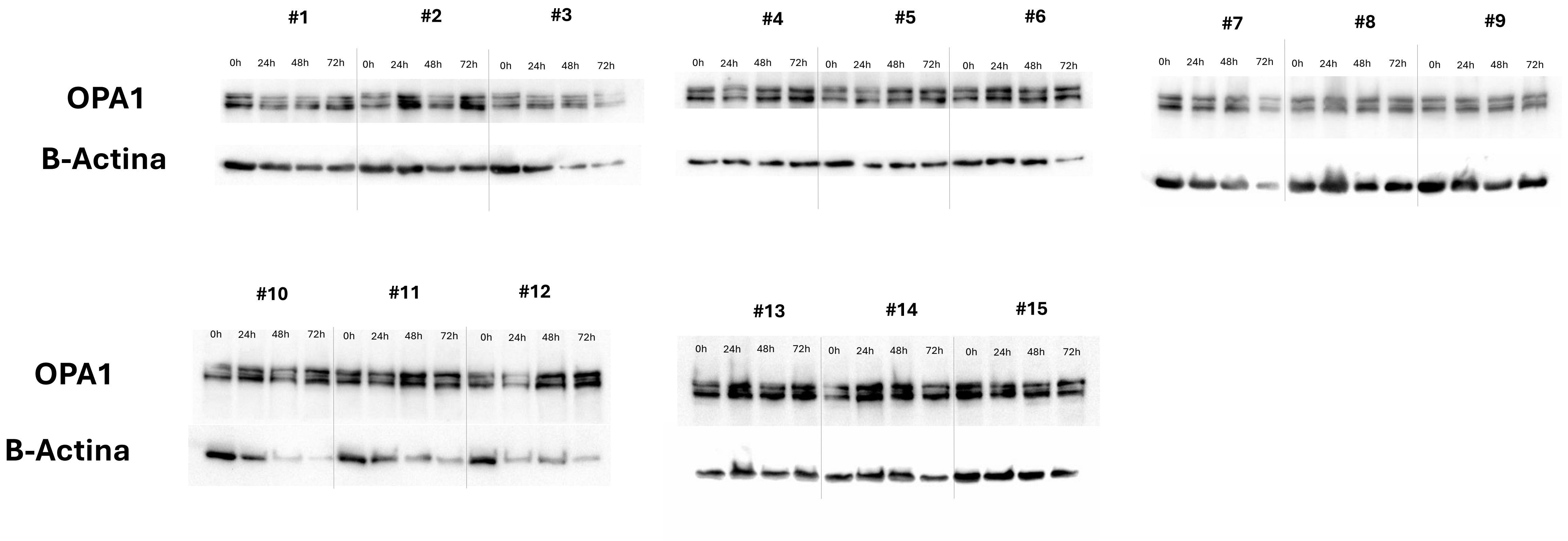
#1
#2
#3
#4
#5
#6
#7
#8
#9
#10
#11
#12
#13
#14
#15
0h
24h
48h
72h
OPA1
B-Actina

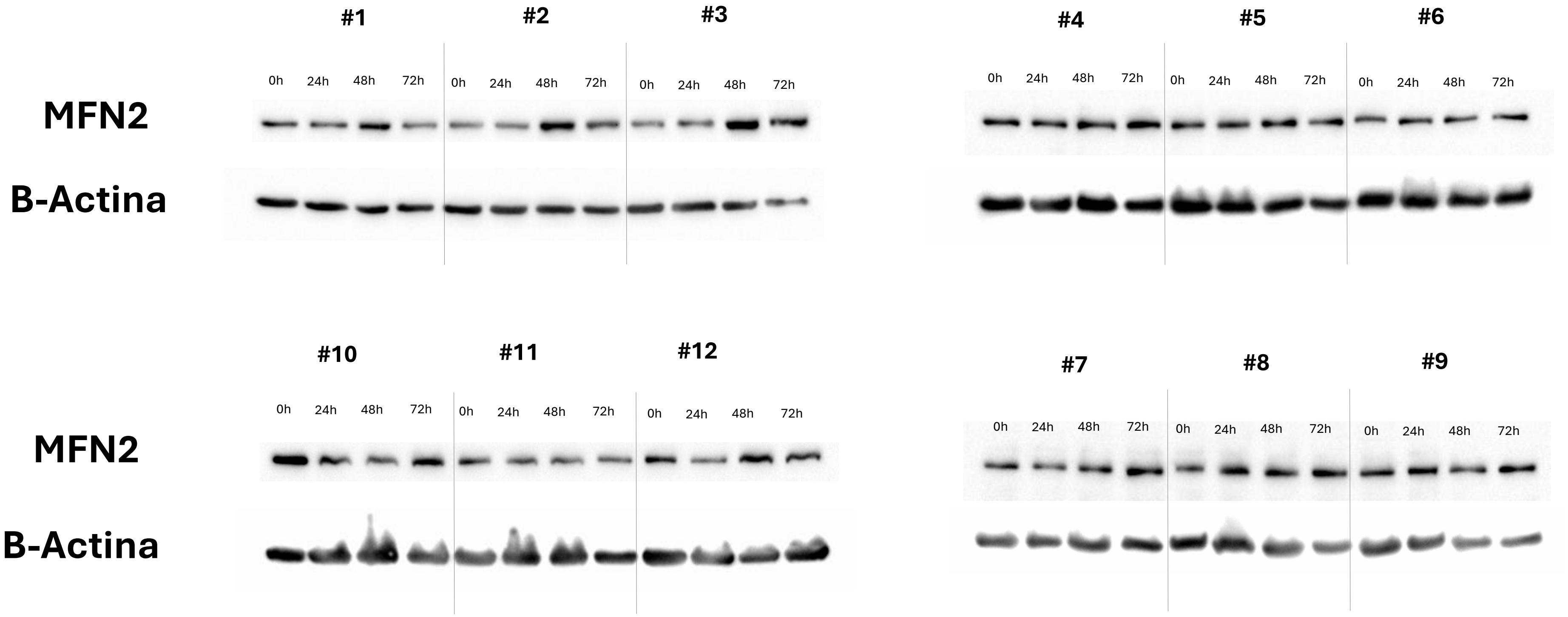
#1
#2
#3
#4
#5
#6
0h
24h
48h
72h
MFN2
B-Actina
#10
#11
#12
#7
#8
#9

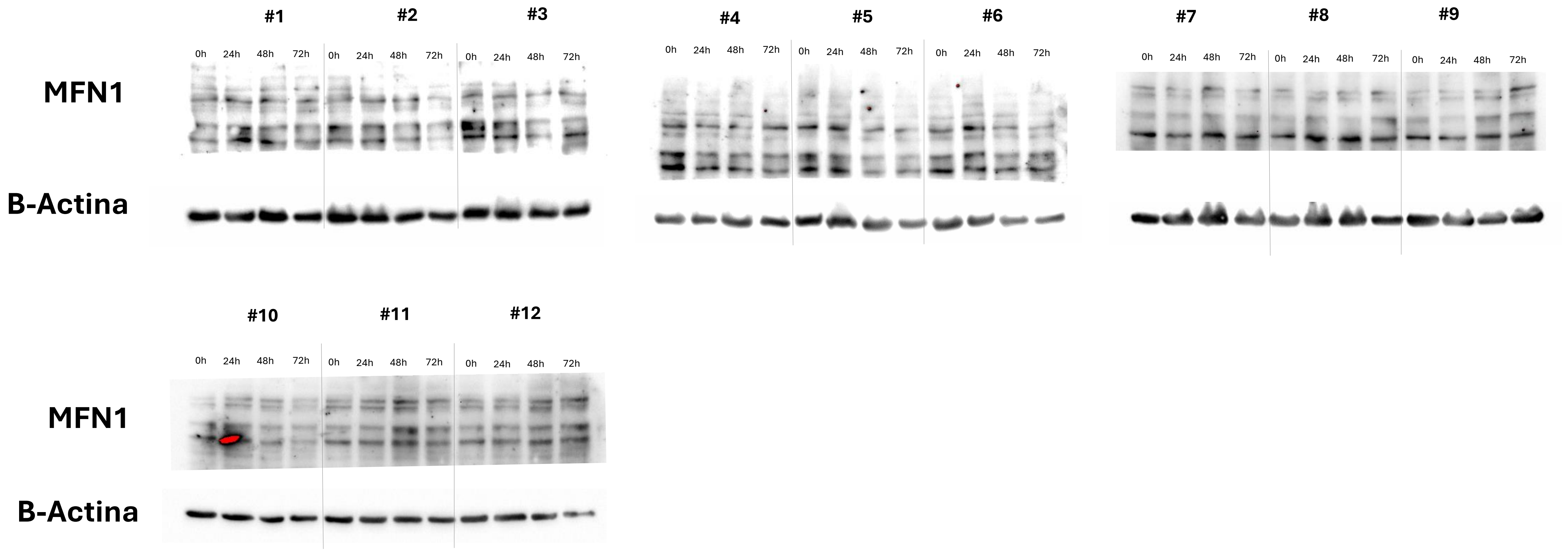

#1
#2
#3
#4
#5
#6
#7
#8
#9
0h
24h
48h
72h
MFN1
B-Actina
#10
#11
#12
0h
24h
48h
72h
MFN1
B-Actina

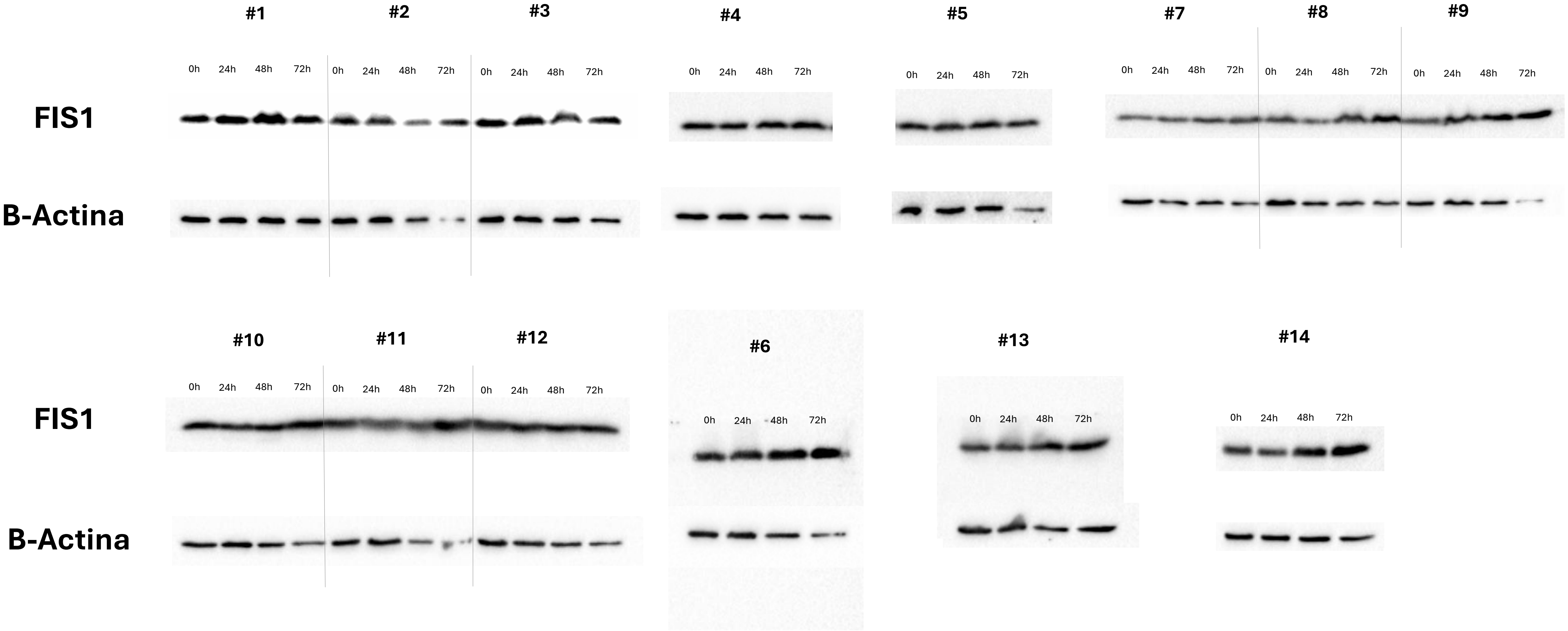
FIS1
B-Actina
#1
#2
#3
#4
#5
#7
#8
#9
0h 24h 48h 72h 0h 24h 48h 72h 0h 24h 48h 72h
0h 24h 48h 72h
0h 24h 48h 72h
0h 24h 48h 72h 0h 24h 48h 72h 0h 24h 48h 72h
FIS1
B-Actina
#10
#11
#12
#6
#13
#14
0h 24h 48h 72h 0h 24h 48h 72h 0h 24h 48h 72h
0h 24h 48h 72h
0h 24h 48h 72h
0h 24h 48h 72h

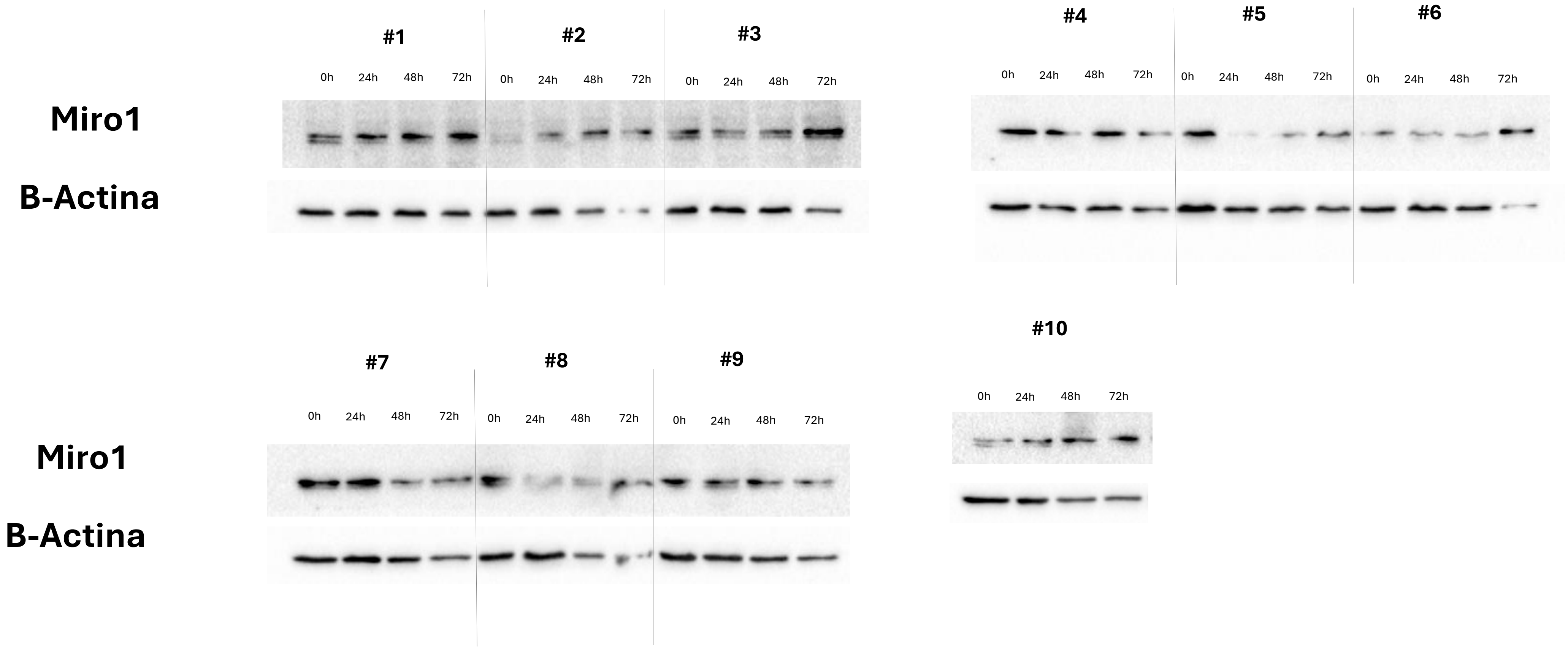
Miro1
B-Actina
#1
#2
#3
0h 24h 48h 72h 0h 24h 48h 72h 0h 24h 48h 72h
#4
#5
#6
0h 24h 48h 72h 0h 24h 48h 72h 0h 24h 48h 72h
Miro1
B-Actina
#7
#8
#9
0h 24h 48h 72h 0h 24h 48h 72h 0h 24h 48h 72h
#10
0h 24h 48h 72h

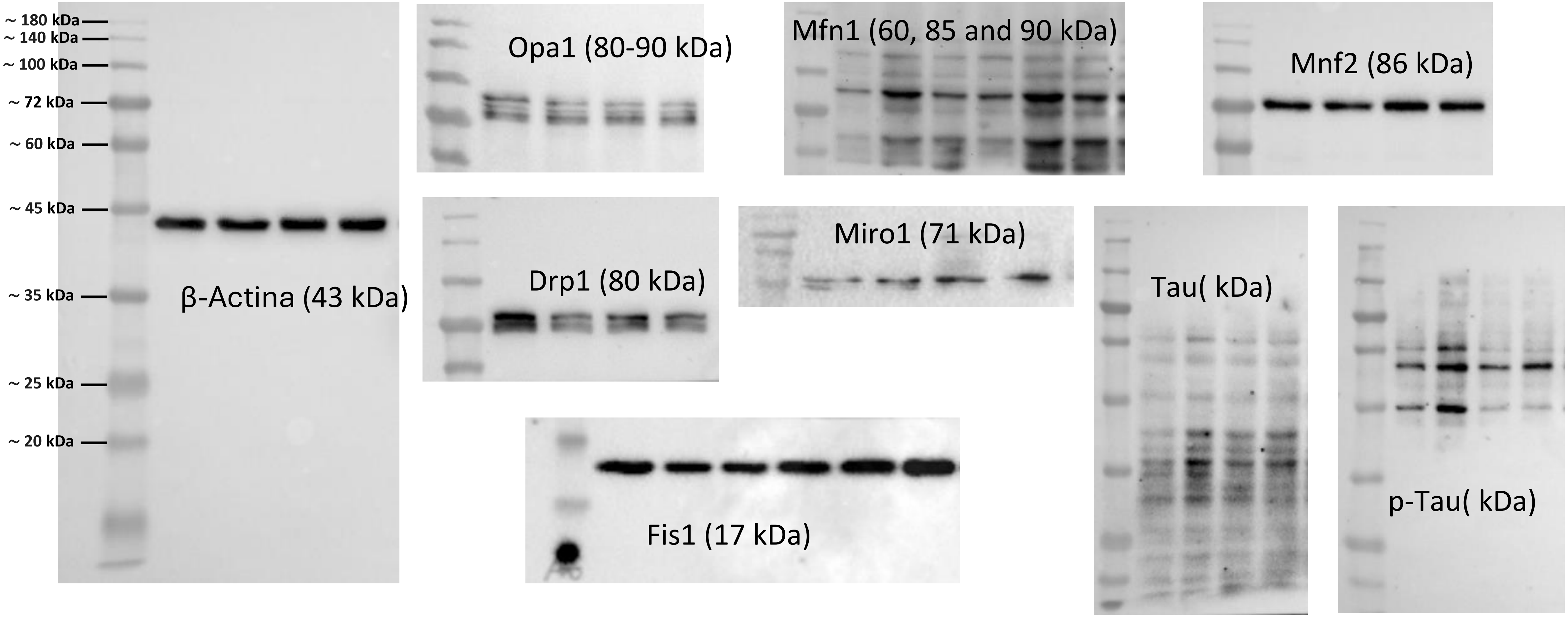
~ 180 kDa
~ 140 kDa
~ 100 kDa
~ 72 kDa
~ 60 kDa
~ 45 kDa
~ 35 kDa
~ 25 kDa
~ 20 kDa
β-Actina (43 kDa)
Opa1 (80-90 kDa)
Mfn1 (60, 85 and 90 kDa)
Mnf2 (86 kDa)
Drp1 (80 kDa)
Miro1 (71 kDa)
Tau( kDa)
p-Tau( kDa)
Fis1 (17 kDa)
