# Supplementary figures and images for: The Synthetic Cannabinoid ADB-FUBINACA Disrupts Mitochondrial Morphology and Dynamics during Neuronal Differentiation of NG108-15 Cells
Source: Mol Neurobiol. 2026 Jan 21;63(1):382. doi: 10.1007/s12035-026-05699-x (PMC12823749; doi:10.1007/s12035-026-05699-x)

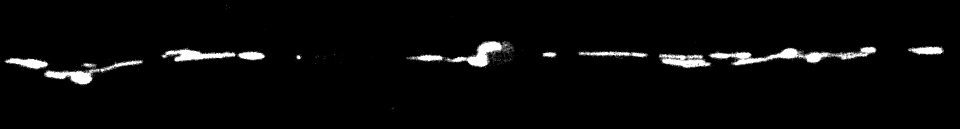

Supplement: Supplementary file 3 — Supplementary file3 (GIF 1850 KB) [file 12035_2026_5699_MOESM3_ESM.gif]

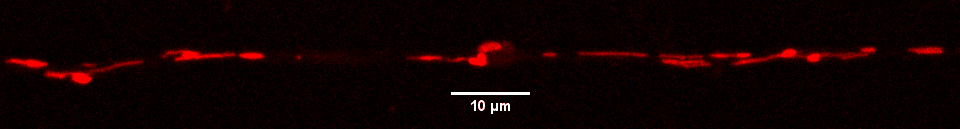

Supplement: Supplementary file 4 — Supplementary file4 (GIF 25394 KB) [file 12035_2026_5699_MOESM4_ESM.gif]

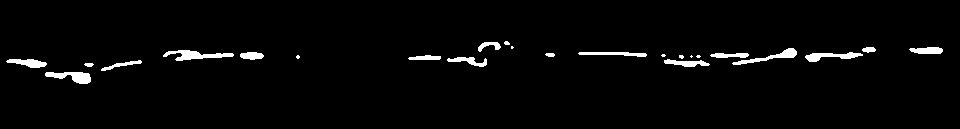

Supplement: Supplementary file 5 — Supplementary file5 (GIF 586 KB) [file 12035_2026_5699_MOESM5_ESM.gif]

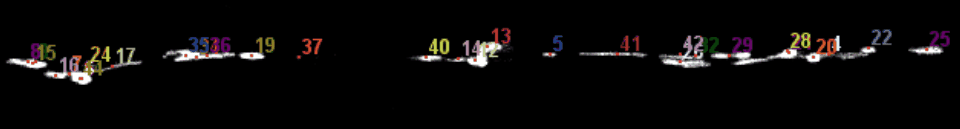

Supplement: Supplementary file 6 — Supplementary file6 (GIF 6207 KB) [file 12035_2026_5699_MOESM6_ESM.gif]

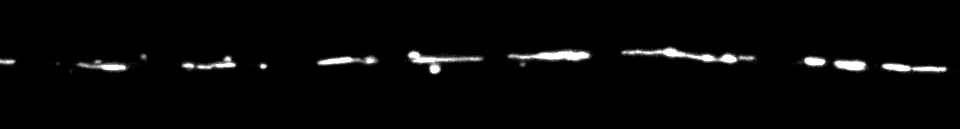

Supplement: Supplementary file 7 — Supplementary file7 (GIF 3296 KB) [file 12035_2026_5699_MOESM7_ESM.gif]

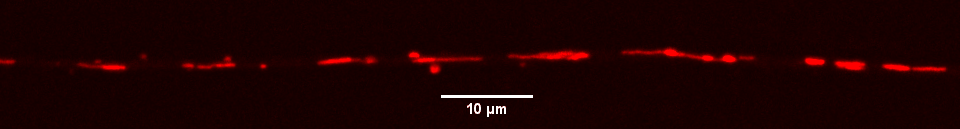

Supplement: Supplementary file 8 — Supplementary file8 (GIF 23259 KB) [file 12035_2026_5699_MOESM8_ESM.gif]

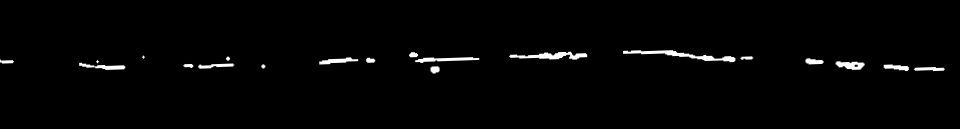

Supplement: Supplementary file 9 — Supplementary file9 (GIF 781 KB) [file 12035_2026_5699_MOESM9_ESM.gif]

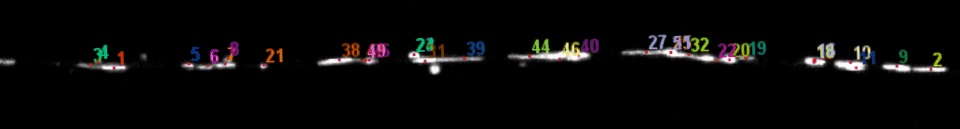

Supplement: Supplementary file 10 — Supplementary file10 (GIF 7212 KB) [file 12035_2026_5699_MOESM10_ESM.gif]
